# Recent Advances in Biomimetic Hydrogels for Bioelectronics and Human–Machine Interactions

**DOI:** 10.3390/gels12090772

**Published:** 2026-08-28

**Authors:** Tianzeng Hong, Zhenpeng Han, Buwei Zheng, Shihao Lu, Yiwei Tan, Baojin Chen, Yanchao Mao

**Affiliations:** 1 School of Science, Henan Institute of Technology, Xinxiang 453003, China; 2 Key Laboratory of Materials Physics of Ministry of Education, School of Physics, Zhengzhou University, Zhengzhou 450001, China

**Keywords:** biomimetic hydrogels, bioelectronics, human–machine interfaces, bioinspired design

## Abstract

Biomimetic hydrogels have emerged as versatile bioelectronic interface materials for bioelectronics and human–machine interfaces (HMIs), enabling mechanically compliant and multifunctional interactions between electronic devices and biological tissues. Inspired by the structures and functions of biological systems, these hydrogels incorporate tissue-like mechanics, efficient ionic/electronic transport, robust wet adhesion, and environmental adaptability within hydrated polymer networks, enabling stable bioelectronic interfaces. This review first categorizes biomimetic hydrogels into polymer-based hydrogels, carbon–polymer composites, and metal–polymer composites, with emphasis on their structural features and functional properties. We then discuss biomimetic strategies for regulating charge transport, mechanical performance, and interfacial adhesion through structural and molecular engineering, highlighting how biomimetic principles are translated into material properties. Finally, representative applications in electrophysiological monitoring, biochemical sensing, gesture recognition, and robotic control are discussed to establish the link between biomimetic material design and device functionality. Overall, this review highlights the design principles that connect biological inspiration to material properties and bioelectronic functions, providing a framework for the development of hydrogel-based biointerfaces for advanced bioelectronics and HMIs.

## 1. Introduction

The integration of materials science, bioengineering, and electronics has enabled the development of bioelectronic technologies that support stable and efficient communication with biological tissues [1,2,3,4,5,6,7,8]. These technologies enable continuous physiological monitoring, targeted therapy, and intuitive human–machine interactions (HMIs), which in turn require bioelectronic interfaces with high mechanical compliance, efficient ionic/electronic transport, and long-term operational stability [9,10,11,12,13,14]. However, conventional metallic and rigid silicon-based electrodes exhibit a pronounced mechanical mismatch with soft tissues, resulting in interfacial stress, inflammatory responses, and progressive deterioration of recording and stimulation performance during prolonged use [15,16,17]. Hydrogels have therefore attracted considerable interest as bioelectronic interface materials because of their tissue-like mechanical compliance, intrinsic biocompatibility, and ionic conductivity [18,19,20,21,22]. Nevertheless, their practical applications remain limited by insufficient electrical conductivity, inadequate mechanical durability, and unstable wet adhesion, underscoring the need to simultaneously address charge transport, mechanical performance, and interfacial stability [10,23,24,25,26].

Biomimetic design strategies provide an effective route to overcome these limitations by translating structural and functional principles from biological systems into hydrogel architectures [27,28,29,30,31,32,33,34]. Representative examples include skin-inspired gradient architectures for multifunctional sensing [28,35], cartilage-inspired sacrificial networks for enhanced toughness [36,37], and mussel-inspired catechol chemistry for robust wet adhesion [38,39,40,41]. Rather than referring simply to tissue-like properties, biomimetic design in this context involves the translation of specific biological structures, interactions, or functions into material architectures that regulate device-relevant properties. To establish a consistent framework across different hydrogel systems, we organize biomimetic design principles into three complementary and interrelated levels: (i) molecular-level engineering, which regulates charge transport and molecular interactions through mechano-ionic transduction, zwitterionic chemistry, ion-selective transport, and dynamic bonding; (ii) structural-level design, which draws inspiration from biological architectures such as helical fibers, laminated cuticles, and gradient structures to regulate load transfer, energy dissipation, and conductive pathways; and (iii) interface-level optimization, which addresses wet adhesion, interfacial hydration, antifouling, and tissue integration through strategies such as catechol-mediated interactions, suction-assisted attachment, and dry-state interfacial crosslinking. This framework provides a common basis for comparing polymer-, carbon-, and metal-based hydrogel systems and underscores how distinct material platforms can adopt convergent biomimetic strategies to address shared challenges in transport, mechanical, and interfacial performance.

In parallel, advances in polymer chemistry, functional nanomaterials, and fabrication technologies have further broadened the functional capabilities of hydrogel bioelectronics. Conductive polymers, carbon nanomaterials (e.g., graphene, carbon nanotubes (CNTs)), and metal-based conductive components (e.g., silver nanowires (AgNWs), gold nanoparticles, and liquid metal (LM)) have substantially improved the electrical performance, mechanical durability, and functional integration of hydrogel systems [9,17,24,42,43,44,45,46,47]. These advances have enabled biomimetic hydrogels to support a broad range of applications, including electrophysiological monitoring, biochemical sensing, gesture recognition, and robotic control, demonstrating considerable potential for wearable and implantable bioelectronics [26,48,49,50,51,52,53]. Despite these advances, achieving the simultaneous optimization of electrical performance, tissue-matched mechanics, interfacial stability, and long-term biocompatibility remains a major challenge. Further translation toward practical applications requires scalable manufacturing, durable operation under physiological conditions, and the integration of hydrogel biointerfaces with wireless communication, self-powered systems, and intelligent data processing [54,55,56,57].

**Figure 1:**
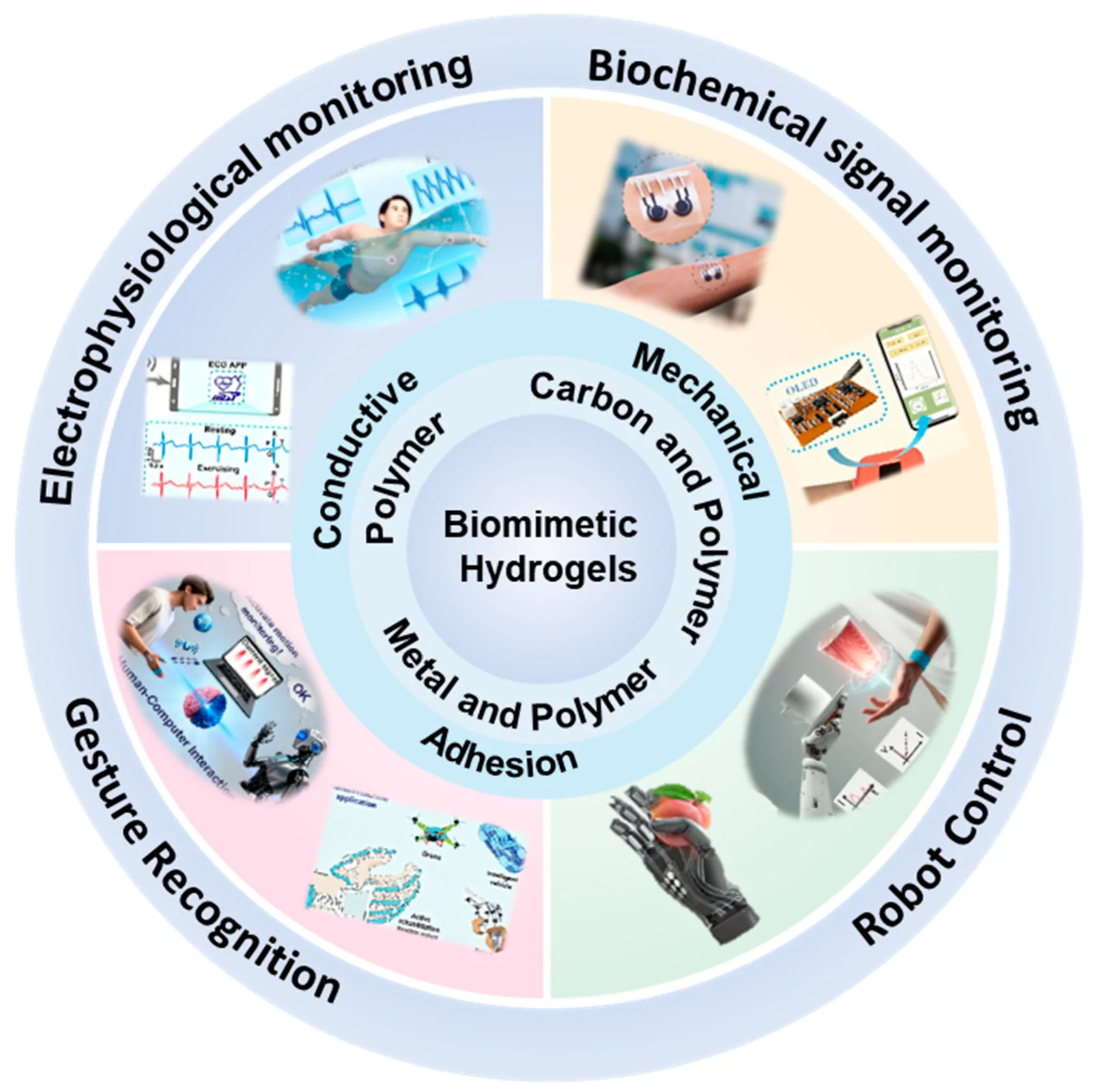
Recent progress of biomimetic hydrogels for bioelectronics and human–machine interactions. Electrophysiological Monitoring: Reproduced with permission [58]. Copyright 2024, Wiley. Reproduced with permission [59]. Copyright 2024, Wiley. Biochemical signal monitoring: Reproduced with permission [22]. Copyright 2026, Wiley. Reproduced with permission [53]. Copyright 2026, Wiley. Robot Control: Reproduced with permission [60]. Copyright 2026, Springer Nature. Reproduced with permission [61]. Copyright 2024, Wiley. Gesture Recognition: Reproduced with permission [62]. Copyright 2026, Springer Nature. Reproduced with permission [63]. Copyright 2026, American Chemical Society.

While several recent reviews have summarized conductive hydrogels for bioelectronics and HMIs, this review emphasizes the translation of biomimetic principles into material design and bioelectronic functions. We adopt a design-oriented framework that links biological inspiration, material design, functional properties, and device performance, with a focus on electrical conductivity, mechanical compliance, and interfacial adhesion across electrophysiological monitoring, biochemical sensing, gesture recognition, and robotic control. This framework enables comparison of how different biomimetic strategies translate into material properties and device-level functions. Accordingly, this review discusses recent advances in biomimetic hydrogels for bioelectronics and HMIs from three complementary aspects: material systems, biomimetic design strategies, and representative applications (Figure 1). We first summarize polymer-based hydrogels including carbon–polymer and metal–polymer composite hydrogels, focusing on their material characteristics and conductive mechanisms. Biomimetic strategies for regulating charge transport, mechanical performance, and interfacial adhesion are then discussed, followed by representative applications in electrophysiological monitoring, biochemical sensing, gesture recognition, and robotic control. Finally, current challenges and future perspectives are presented with an emphasis on multifunctional integration, scalable manufacturing, and intelligent bioelectronic systems. By linking biomimetic principles with application requirements, this review highlights key design considerations for developing next-generation hydrogel biointerfaces.

## 2. Types of Hydrogel Materials

The composition and network architecture of hydrogels critically influence their electrical behavior, mechanical compliance, and biointerfacial properties, which collectively determine their suitability for bioelectronic and HMI applications [64,65,66]. Variations in polymer chemistry, conductive components, and network organization regulate charge transport and mechanical properties while also affecting interfacial stability, environmental tolerance, and biocompatibility [67,68,69]. Therefore, the rational design of hydrogel compositions and network architectures is essential for developing biointerfaces with balanced electrical, mechanical, and interfacial properties for wearable and implantable devices. According to their constituent materials and conductive components, biomimetic conductive hydrogels can be broadly categorized into three categories: polymer-based hydrogels, carbon–polymer composite hydrogels, and metal–polymer composite hydrogels. Each class presents distinct trade-offs among electrical conductivity, mechanical robustness, interfacial adhesion, and biocompatibility. Polymer-based hydrogels generally provide superior biocompatibility and tissue-like compliance but relatively low conductivity, whereas carbon–polymer composites offer improved conductivity and mechanical robustness but may raise concerns regarding nanomaterial biocompatibility and long-term stability. Metal–polymer composites can achieve high electrical conductivity but may be limited by filler–matrix debonding under repeated deformation and limited biodegradability. Thus, optimizing one property often compromises other properties, highlighting the need for hybrid architectures and multifunctional composite designs that integrate complementary material characteristics. A comparative summary of their conductivity, mechanical properties, adhesion, key advantages and limitations, and representative applications is provided in Table 1. These distinctions provide a basis for understanding how different material platforms are subsequently engineered to address their specific electrical, mechanical, and interfacial limitations.

### 2.1. Polymer-Based Hydrogels

Polymer-based hydrogels represent one of the most widely studied hydrogel platforms for bioelectronics owing to their excellent biocompatibility, tunable network architectures, and tissue-like mechanical compliance [70,71,72]. However, their practical applications are often limited by low electrical conductivity, insufficient mechanical robustness, dehydration-induced functional degradation, and poor environmental stability [18,72,73,74]. Recent biomimetic approaches have sought to address these limitations by integrating conductive architectures, hydration regulation, zwitterionic chemistry, and biomineralization into polymer networks to enhance electrical transport, mechanical resilience, and operational stability.

**Figure 2:**
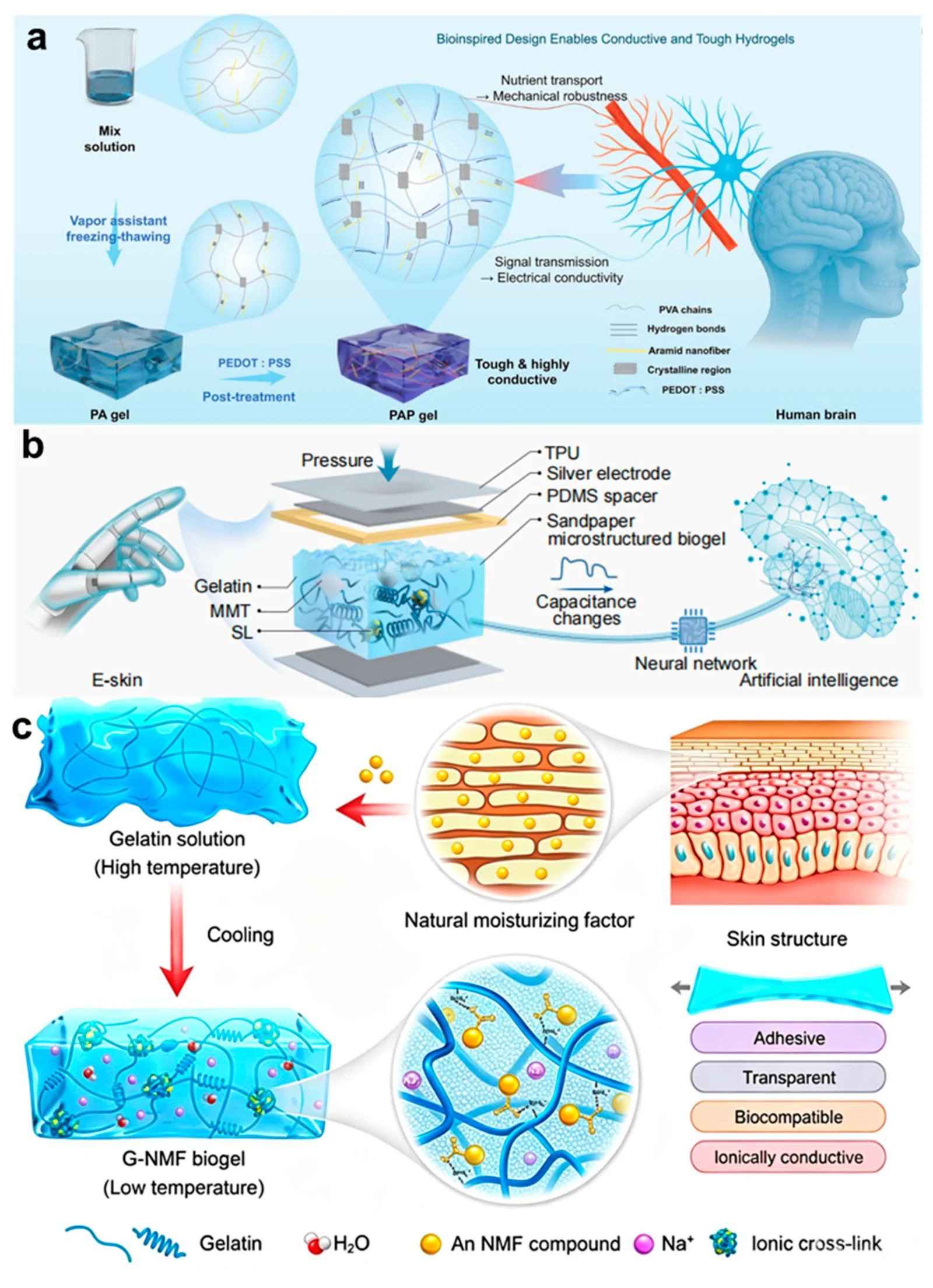
Fabrication strategies and functional demonstrations of polymer-based hydrogels. (**a**) Bioinspired design and fabrication process of the PAP hydrogel [75]. (**b**) Schematic illustration of a skin-inspired gelatin-based biomimetic hydrogel with hierarchical structure, enhanced mechanical properties, and tactile sensing performance [60]. (**c**) Schematic diagram of the preparation process of skin-inspired all-natural biological gel [76].

Bioinspired regulation of molecular interactions and network architectures has substantially improved the electrical, mechanical, and sensing performance of polymer hydrogels. These strategies operate across different structural scales to regulate conductive pathways, hydration states, and mechanical energy dissipation. Inspired by the interconnected organization of biological transport networks, Liu et al. developed a bicontinuous PVA/aramid nanofiber/poly(3,4-ethylenedioxythiophene):poly(styrene sulfonate) (PEDOT:PSS) hydrogel with conductive and reinforcing pathways, achieving high conductivity while maintaining excellent flexibility (Figure 2a) [75]. This architecture helps reconcile the otherwise competing requirements of electrical transport and mechanical compliance. For naturally derived polymers, Lan et al. introduced sodium pyrrolidone carboxylate (PCA-Na) into gelatin to regulate water retention, ionic transport, and network stability, enabling reliable electrophysiological recording under long-term operation (Figure 2b) [60]. By further incorporating sodium lactate and montmorillonite nanosheets, they constructed a skin-inspired biogel with enhanced mechanical robustness and environmental adaptability for tactile sensing [76]. Building on the role of hydration regulation, Wu et al. developed a zwitterionic hydrogel inspired by the hydration mechanism of the stratum corneum that selectively retains bound water and suppresses dehydration (Figure 2c). Incorporation of a triple-crosslinked network further improved environmental tolerance and self-healing capability, enabling reliable wearable sensing under varying conditions [70]. Beyond regulating conductivity and hydration, biomineralization provides an effective strategy for enhancing the mechanical robustness of polymer hydrogels. Inspired by the impact-resistant architecture of the mantis shrimp dactyl club, Tan et al. incorporated enzyme-induced calcium phosphate nanoparticles into a polymer network, in which multiscale mineral–polymer interactions promoted efficient energy dissipation through crack deflection and nanoparticle reinforcement [77]. Together, these examples demonstrate how biomimetic regulation of conductive pathways, hydration, and multiscale architectures can address distinct but interconnected electrical, mechanical, and environmental requirements in polymer hydrogels for wearable and implantable bioelectronics.

A critical analysis of polymer-based hydrogels reveals fundamental trade-offs among electrical conductivity, mechanical durability, and environmental stability. Strategies that exploit controlled failure mechanisms enhance energy dissipation and sensing sensitivity but may compromise long-term reliability. Conversely, approaches that reinforce network continuity improve stability but often yield moderate sensitivity. Under repeated mechanical deformation, progressive disruption of conductive pathways can lead to signal drift and resistance changes, particularly when the network lacks sufficient structural recovery. Dehydration remains another persistent challenge, as water loss can induce network contraction, alter ion transport, and degrade sensing performance. In addition, variations in crosslinking density, conductive-component dispersion, and processing conditions can compromise batch-to-batch reproducibility. These limitations highlight the need for standardized long-term stability testing protocols and improved manufacturing consistency before clinical translation.

### 2.2. Carbon and Polymer Composite Hydrogels

Carbon–polymer composite hydrogels combine the mechanical compliance of polymer networks with the high electrical conductivity of carbon nanomaterials, offering versatile platforms for constructing stable and efficient bioelectronic interfaces [9,78,79,80,81,82]. Carbon nanomaterials markedly improve electrical transport and mechanical robustness while maintaining the intrinsic compliance of hydrogel matrices [36,83]. However, the strong interparticle interactions and surface-energy effects can induce aggregation, leading to heterogeneous conductive networks and reduced structural reliability. Recent biomimetic strategies have sought to address these limitations by improving nanomaterial dispersion, interfacial interactions, and conductive pathway formation through surface functionalization and hierarchical structural engineering.

**Figure 3:**
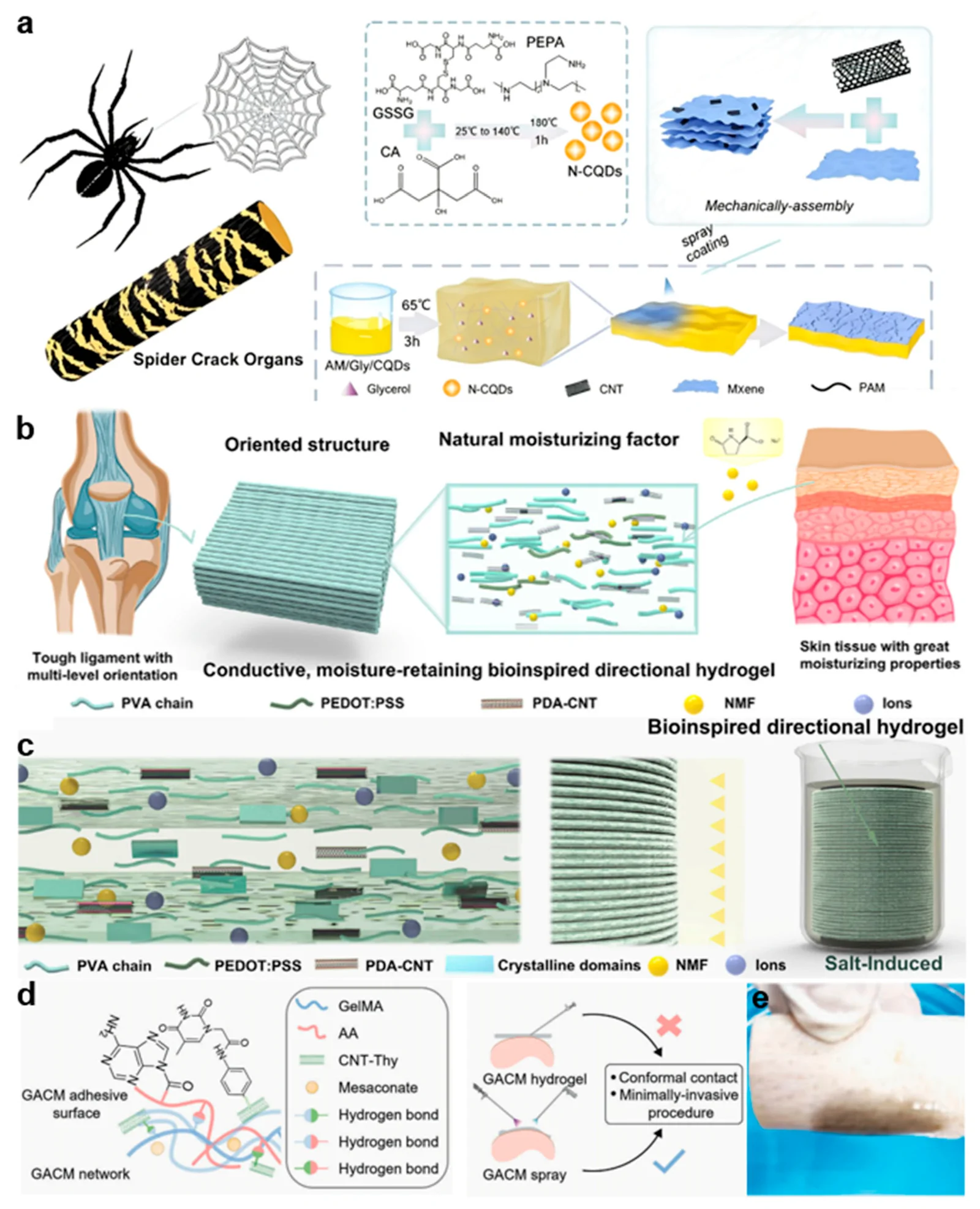
Fabrication strategies and functional demonstrations of carbon and polymer composite hydrogels. (**a**) Spider slit-inspired crack structure with N-CQDs and MXene/CNT for high-sensitivity mechanosensing [84]. (**b**) Ligament-inspired anisotropic hydrogel with aligned conductive networks and PCA-Na moisturizing for enhanced mechanical and electrical properties [36]. (**c**) Shear/stretch-induced alignment and salt-induced crystallization to construct directionally ordered, robust nanofibril networks [36]. (**d**) Nucleobase-inspired adhesive conductive spray with gelatin–adenine–CNT networks for in situ conformal adhesion and nerve repair [83]. (**e**) Photograph showing strong skin adhesion of the sprayable hydrogel [83].

Inspired by spider slit organs, Jiang et al. developed a crack-enabled conductive hydrogel by depositing a CNT/MXene conductive layer onto a polyacrylamide hydrogel containing fluorescent carbon quantum dots (CQDs) (Figure 3a) [84]. The controlled evolution of microcracks generated large resistance changes over a broad strain range, while glycerol-mediated hydrogen bonding enhanced water retention and antifreeze capability. The incorporation of CQDs further enabled optical visualization of deformation alongside electrical signal transduction, providing additional sensing information beyond conventional resistive detection. Surface functionalization represents an effective strategy for improving the compatibility between carbon nanomaterials and hydrogel matrices. As a representative example, Wang et al. functionalized CNTs with polydopamine to improve hydrophilicity and strengthen interfacial interactions within the polymer network (Figure 3b) [36]. Inspired by tissue anisotropy and skin hydration regulation, they further fabricated a directionally structured hydrogel through flow-induced alignment followed by sodium PCA treatment. The aligned conductive network simultaneously improved tensile strength, fracture resistance, and electrical stability, enabling reliable motion recognition through machine-learning-based signal analysis. Beyond surface modification, hierarchical structural engineering can further regulate conductive pathways and mechanical responses in carbon–polymer composite hydrogels.

Beyond wearable sensing, carbon–polymer composite hydrogels have also demonstrated considerable potential for implantable bioelectronics. Wang et al. incorporated thymine-functionalized CNTs into a gelatin network to construct an adhesive conductive hydrogel (Figure 3d,e). CNT functionalization improved dispersion and enhanced interfacial interactions through multiple hydrogen bonds. The resulting hydrogel provided stable conductive pathways and continuously released mesaconate to suppress inflammation and promote Schwann cell migration, facilitating peripheral nerve regeneration. Validation in rat and canine models further supported the potential of this strategy for biointegrated neural interfaces [83]. Overall, the performance of carbon–polymer composite hydrogels depends strongly on the dispersion of conductive nanomaterials, interfacial interactions, and the architecture of conductive networks. Coordinated regulation of these factors enables a balanced electrical conductivity, mechanical robustness, and environmental stability, making them promising materials for wearable electronics, implantable neural interfaces, and HMI applications.

### 2.3. Metal and Polymer Composite Hydrogels

Metal–polymer composite hydrogels combine the high electrical conductivity of metallic fillers with the mechanical compliance of polymer networks, offering conductive and mechanically adaptive interfaces for bioelectronic applications [24,85,86,87,88,89,90]. Nevertheless, maintaining stable conductive pathways under repeated deformation and strong filler–matrix integration remain major challenges. Recent biomimetic strategies have sought to address these limitations through interfacial engineering, anisotropic structural design, and dynamic coordination chemistry, improving electrical stability, mechanical reliability, and operational durability.

**Figure 4:**
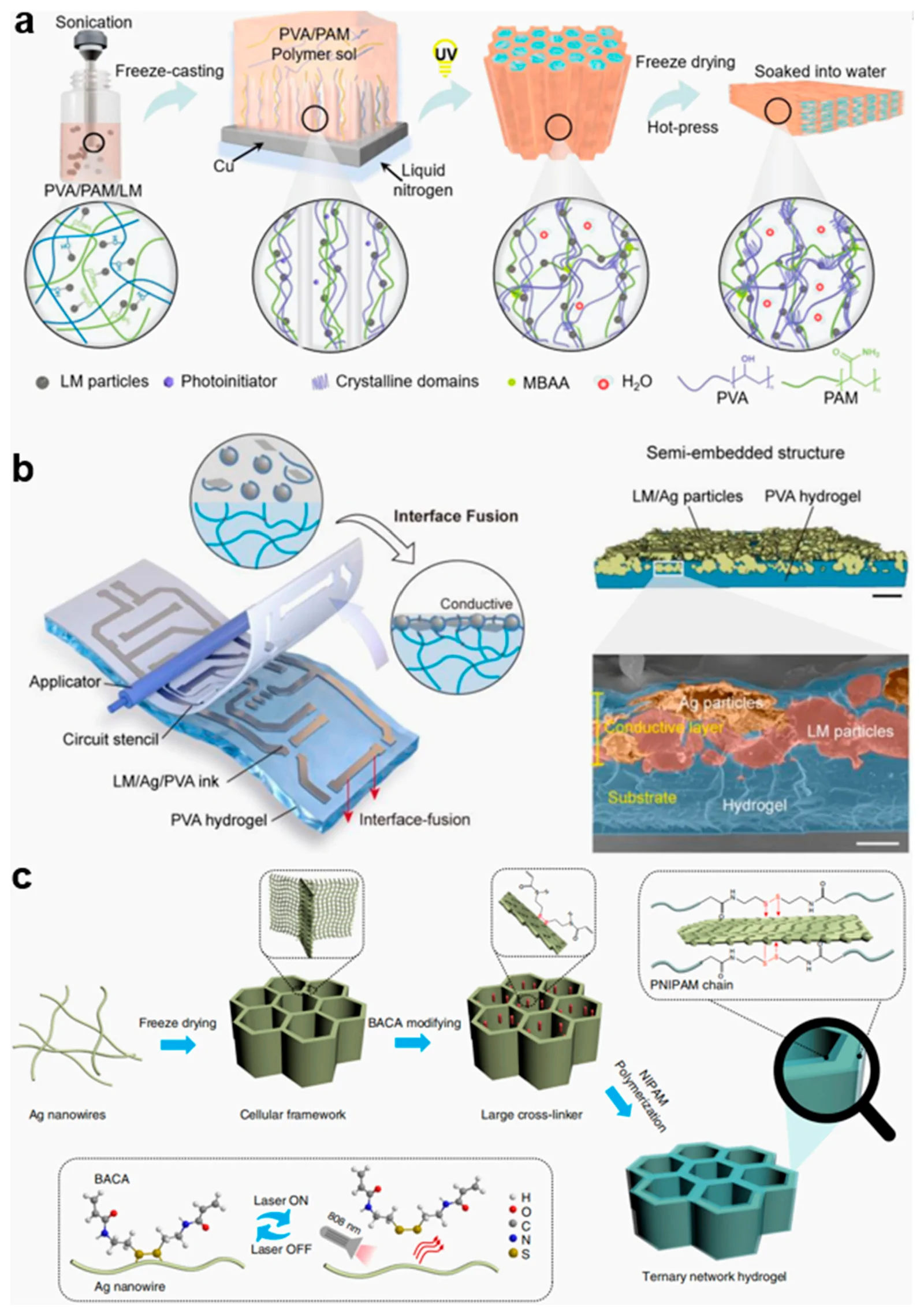
Fabrication strategies and functional demonstrations of metal–polymer composite hydrogels. (**a**) Schematic illustration of anisotropic DN-PPL hydrogel with aligned dual networks and LM particles for enhanced toughness and fatigue resistance [92]. (**b**) Schematic illustration of MPEH fabricated by interface-fusion printing of LM/Ag/PVA composites, enabling highly adhesive and strain-insensitive conductive pathways [91]. (**c**) Schematic illustration of AATN hydrogel based on AgNWs aerogel macro-crosslinkers and dynamic Ag-S coordination for high stretchability, stable electromechanical performance, and self-healing capability [93]. Scale bar, 5 cm.

Interfacial engineering provides an effective strategy for constructing mechanically stable conductive networks. Jiao et al. developed an interface-fusion printing strategy to construct a metal-particle semi-embedded hydrogel by introducing LM and silver microparticles into a PVA-based ink (Figure 4b) [91]. Cooperative dehydration induced cross-interfacial crystallization, generating strong bonding between the conductive layer and hydrogel substrate while suppressing delamination during repeated deformation. The resulting hydrogel maintained stable electrical and mechanical performance under cyclic stretching, impact loading, and underwater operation. Beyond interfacial engineering, structural regulation further improves the mechanical reliability of metal–polymer composites. Inspired by the anisotropic organization of load-bearing tissues, Cui et al. fabricated hierarchically aligned double-network hydrogels reinforced with deformable LM microdroplets through directional freeze-casting (Figure 4a) [92]. The aligned architecture facilitated crack deflection, fiber bridging, and multiscale energy dissipation, thereby improving fatigue resistance, mechanical compliance, and strain-sensing reliability.

Dynamic coordination chemistry provides a complementary route for developing durable conductive hydrogels. Song et al. infiltrated poly(N-isopropylacrylamide) (PNIPAM) into preassembled AgNW aerogels to construct self-healable conductive networks stabilized by dynamic Ag–thiolate coordination (Figure 4c) [93]. The interconnected conductive framework enabled rapid structural recovery while maintaining stable charge transport during repeated deformation, enabling stretchable and reconfigurable bioelectronic devices. Overall, recent advances demonstrate that the performance of metal–polymer composite hydrogels is governed by the synergistic regulation of filler-matrix interfaces, conductive network architecture, and dynamic molecular interactions. These strategies address distinct failure modes associated with interfacial delamination, mechanical damage, and conductive-network disruption, thereby improving the overall reliability of metal–polymer composite hydrogels. By integrating these complementary design principles, metal–polymer composites can be tailored for wearable bioelectronics, soft robotics, and implantable devices. To facilitate a systematic comparison of representative biomimetic hydrogel systems, Table 2 summarizes their key material and device-level characteristics, including electrical conductivity, stretchability, adhesion strength, durability, and representative application scenarios. The comparison covers the three major material categories discussed in this review and highlights the distinct performance advantages and trade-offs associated with different biomimetic design strategies.

## 3. Biomimetic Design Strategies for Hydrogels

Biological systems offer diverse structural and functional principles for designing hydrogel biointerfaces with integrated electrical, mechanical, and interfacial properties [32,33,40,41,94]. Rather than relying solely on empirical optimization, biomimetic design translates hierarchical architectures, dynamic molecular interactions, and adaptive transport mechanisms found in biological systems into material structures, providing strategies to address the intrinsic trade-offs among charge transport, mechanical robustness, and interfacial stability [16,95,96,97,98,99]. This section discusses recent biomimetic strategies for designing hydrogel biointerfaces for bioelectronics and HMIs from three complementary perspectives: conductive properties, mechanical performance, and interfacial adhesion. Despite these conceptual advantages, translating biomimetic architectures into practical devices introduces distinct manufacturing hurdles. The scalability and cost of biomimetic fabrication vary substantially among different strategies. Interface-fusion printing offers precise spatial patterning of conductive components but requires stringent control over ink rheology, whereas directional freeze-casting can generate well-defined anisotropic architectures but involves relatively lengthy processing steps and may exhibit batch-to-batch variability. Extrusion and solvent-assisted assembly offer greater scalability, although maintaining structural uniformity and functional consistency at high throughput remains challenging. These considerations highlight the persistent gap between laboratory-scale demonstrations and scalable manufacturing, arising from factors such as material cost, process complexity, equipment requirements, and manufacturing consistency.

### 3.1. Biomimetic Strategies for Conductive Properties

Biological tissues achieve efficient bioelectrical signaling through highly organized ion-transport pathways while maintaining mechanical adaptability and stable hydration states. Drawing from biological ion-transport mechanisms, recent biomimetic hydrogels have been engineered to regulate ionic conduction across molecular, nanoscale, and hierarchical levels through mechano-ionic transduction, dynamic conductive networks, anisotropic architectures, and ion-selective transport, thereby enhancing charge transport, sensing sensitivity, and operational stability [100,101,102]. These strategies can be broadly understood through three complementary mechanisms: coupling mechanical deformation with ion redistribution, establishing preferential pathways for directional or selective ion transport, and dynamically reorganizing conductive domains to adapt charge-transport routes.

**Figure 5:**
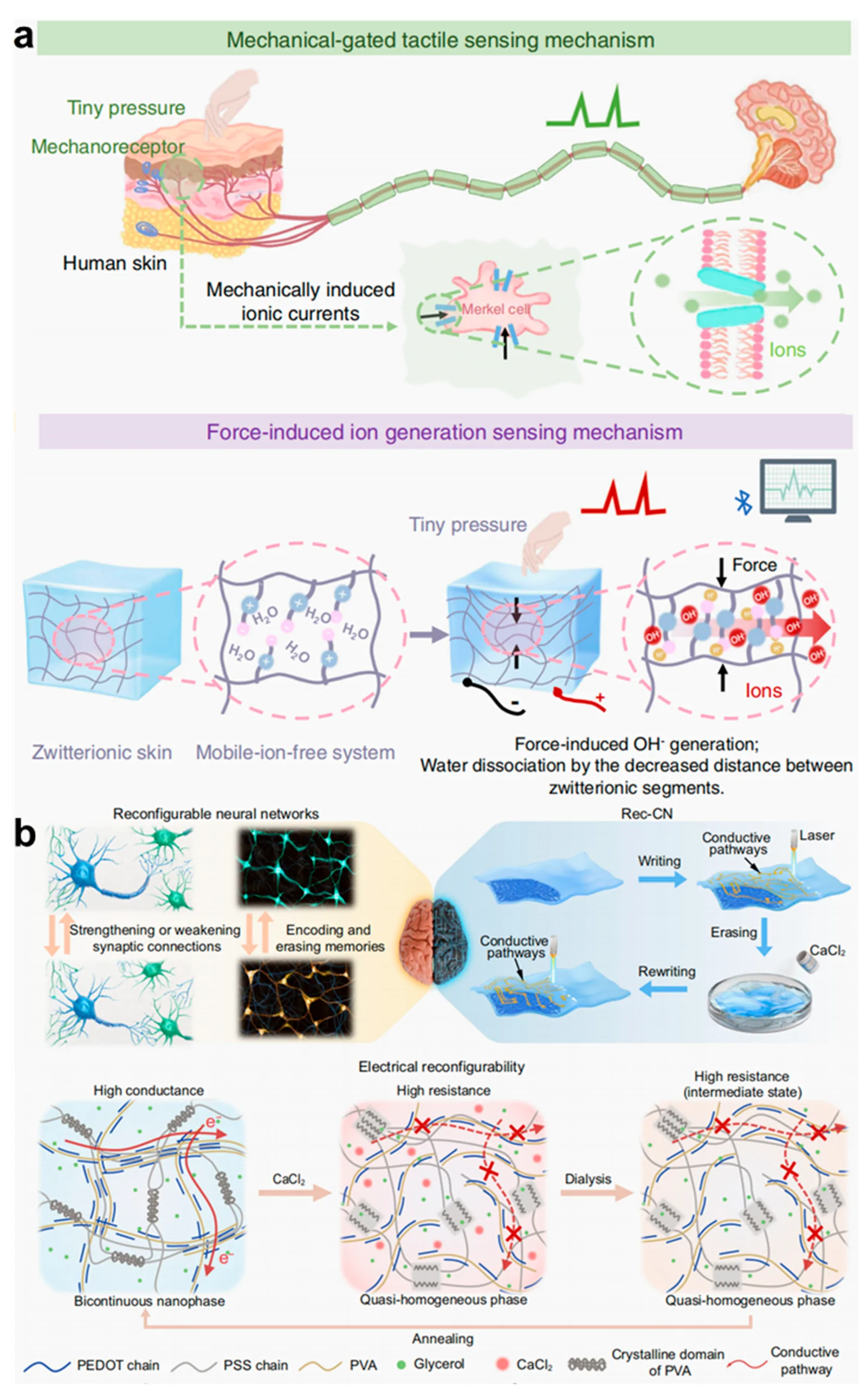
Biomimetic design strategies and functional demonstrations of conductive hydrogels. (**a**) Schematic illustration of a mechanogated ion-channel-inspired strategy, where zwitterionic PCBMA hydrogels generate mobile ions through pressure-induced water dissociation to mimic the mechanosensing mechanism of skin receptors and enable highly sensitive, rapid-response silent-speech recognition [34]. (**b**) Schematic illustration of a neural plasticity-inspired reconfigurable strategy, where CP-PVA organogel-based Rec-CN achieves reversible nanophase regulation through specific ion effects, enabling dynamic patterning, erasing, and rewriting of conductive pathways for reconfigurable electronics with in situ solderability and closed-loop recyclability [103].

Mechano-ionic transduction directly converts mechanical stimuli into electrical signals through deformation-induced ion redistribution. Its sensing performance is closely associated with the concentration of mobile ions and the mobility mismatch between cations and anions, while zwitterionic networks can further enhance the response through their fixed charges and associated ion concentration gradients. Inspired by mechanically gated ion channels in human skin, Xu et al. developed a zwitterionic poly(carboxybetaine methacrylate) (PCBMA) hydrogel in which mechanical deformation promoted water dissociation and generated additional mobile ions through enhanced electrostatic interactions [34]. Meanwhile, the zwitterionic network provided continuous ion-transport pathways, enabling highly sensitive mechano-ionic sensing for wearable silent-speech recognition (Figure 5a). This example illustrates how deformation-induced ion generation and redistribution can directly couple mechanical inputs to ionic electrical outputs.

Dynamic regulation of conductive networks represents another strategy for developing adaptive bioelectronics. In contrast to static conductive pathways, dynamic phase reorganization enables adaptive charge routing through reversible segregation between conductive and polymer-rich domains, conceptually analogous to the plasticity of biological synaptic networks. Inspired by neural synaptic plasticity, Zhong et al. engineered reconfigurable PEDOT:PSS/PVA organogels by exploiting reversible nanophase rearrangement between conductive and polymer-rich domains (Figure 5b) [103]. Dynamic phase reorganization allowed for reversible modulation of charge transport and facilitated laser-patterned circuit fabrication, electrical repair, and material recycling, highlighting the role of dynamic network reorganization in reconfigurable charge transport.

Hierarchical structural organization further improves both charge transport and mechanical reliability. At the structural level, anisotropic architectures establish preferential ion-migration pathways, analogous to the oriented transport channels found in biological excitable tissues, thereby coupling directional conductivity with mechanical reinforcement. Inspired by the anisotropic architectures of biological tissues, Xue et al. fabricated directionally aligned conductive hydrogels through solvent-assisted assembly and ionic crosslinking [41]. The ordered conductive architecture simultaneously enhanced electrical conductivity, tensile strength, and toughness, enabling reliable strain sensing and intelligent material recognition. These results demonstrate that structural anisotropy effectively optimizes conductive pathways while improving mechanical robustness. Selective ion transport represents an additional biomimetic strategy for enhancing piezoionic sensing. Inspired by biological ion-channel selectivity, Fang et al. designed a piezoionic hydrogel by incorporating lithium bis(trifluoromethanesulfonyl)imide (LiTFSI) into a zwitterionic poly(SBMA-co-AM) network [33]. Dipole–ion interactions accelerated ion migration, while the mobility difference between cations and anions amplified the piezoionic response, enabling highly sensitive joint-motion sensing for wearable HMIs.

Collectively, these studies demonstrate that biomimetic regulation of charge transport operates across multiple scales, from molecular ion interactions and selective transport to dynamic conductive networks and hierarchical architectures. By coupling ion redistribution, preferential transport pathways, and adaptive network reorganization, these strategies provide complementary routes to balance charge-transport efficiency, sensing sensitivity, and mechanical reliability in hydrogel biointerfaces.

### 3.2. Biomimetic Strategies for Mechanical Properties

Natural load-bearing tissues achieve high mechanical strength, toughness, and fatigue resistance through multiscale structural organization, which facilitates efficient load transfer and energy dissipation from molecular to macroscopic length scales. Drawing from these principles, biomimetic hydrogels have been designed with aligned fibrous networks, laminated architectures, and molecular nanoconfined structures to enhance strength, toughness, and fatigue resistance, approaching the mechanical characteristics of natural load-bearing tissues [29,30,31,104].

**Figure 6:**
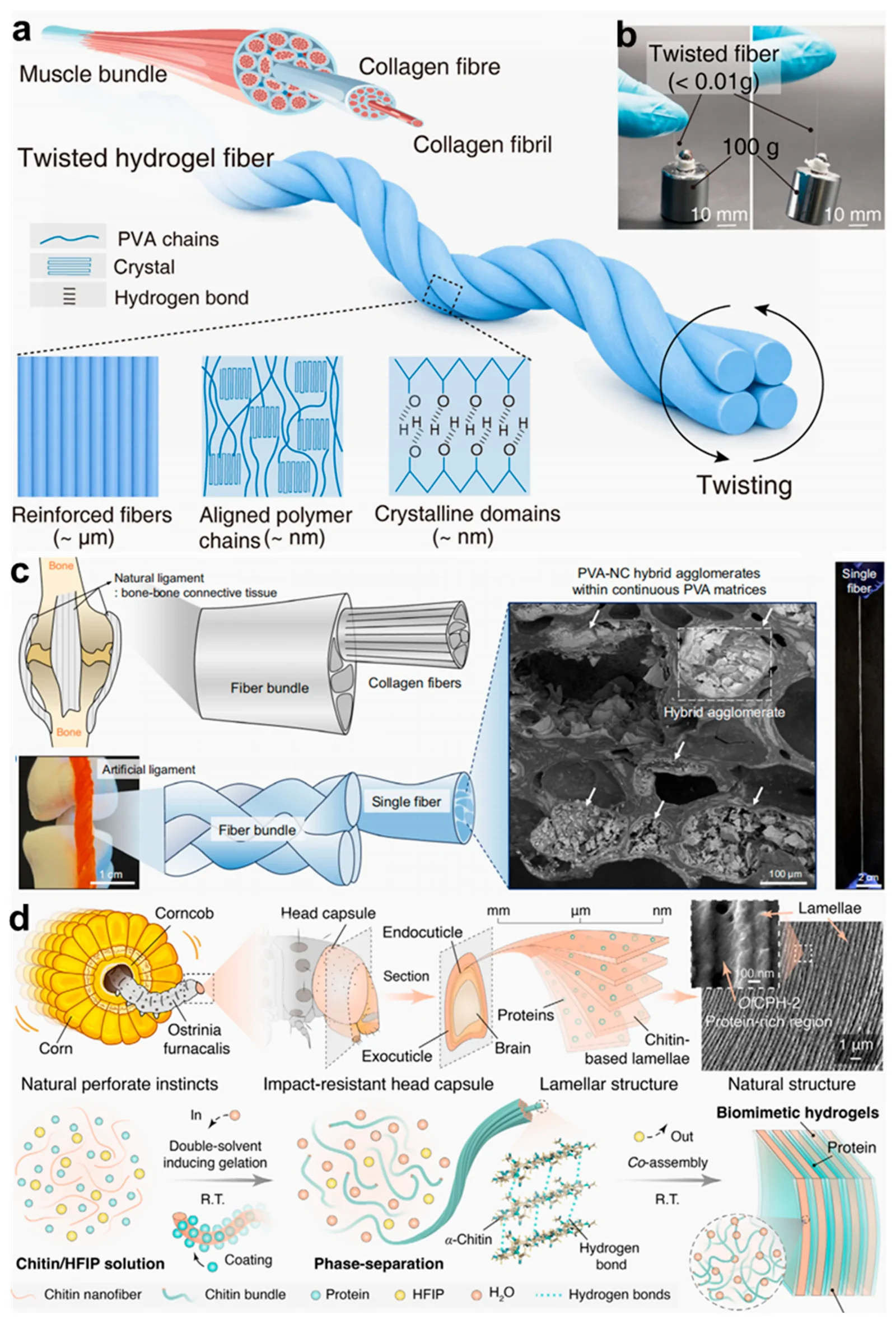
Biomimetic mechanical design strategies and functional demonstrations of hydrogels. (**a**) Schematic illustration of hierarchical muscle-inspired structures and fatigue-resistant hydrogel fibers, ranging from aligned polymer chains and nanocrystals to twisted fiber assemblies, where multiscale structural organization synergistically enhances crack resistance and fatigue tolerance [105]. (**b**) Demonstration of the ultrahigh mechanical strength of twisted hydrogel fibers capable of supporting a load 10,000 times their own weight [105]. (**c**) Schematic illustrations of natural and artificial ligaments with hierarchically aligned fiber structures, together with structural characterization of artificial hydrogel fibers, including cross-sectional SEM images showing PVA-NC hybrid aggregates embedded within continuous PVA matrices and optical images of individual fibers [37]. (**d**) Schematic illustration of the biomimetic design inspired by the impact-resistant head cuticle of Asian corn borer larvae and the fabrication of BM-hydrogel through phase separation-induced co-assembly of chitin and OfCPH-2 into a lamellar hierarchical architecture [101].

Aligned fibrous architectures provide an efficient means of improving mechanical robustness. Inspired by the helical organization of collagen fibers in tendons, Liu et al. fabricated hierarchically twisted PVA hydrogel fibers through sequential stretching, torsion, and thermal annealing (Figure 6a) [105]. The multiscale helical architecture redistributed stress through inter-fiber friction and progressive energy dissipation, thereby enhancing fatigue resistance while maintaining high strength and toughness (Figure 6b). Inspired by the parallel fiber bundles of ligaments, Shi et al. fabricated highly aligned PVA-nanoclay hydrogel fibers through extrusion, freeze–thawing, and salting-out processes (Figure 6c) [37]. Nanoclay not only facilitated fiber formation but also reinforced the polymer network by promoting efficient stress transfer at the polymer–particle interface. The resulting hydrogel exhibited mechanical characteristics similar to ligaments and was assembled into braided strain-sensing structures capable of sustaining high mechanical loads. Laminated architectures provide another structural solution for improving impact resistance and fatigue durability. Inspired by the multilayer organization of insect cuticles, Wu et al. constructed chitin-protein lamellar structures through binary solvent-induced assembly using recombinant structural proteins (Figure 6d) [101]. Progressive deformation of individual lamellae, together with α-helix unfolding within the protein network, effectively dissipated impact energy and suppressed crack propagation, resulting in high resistance to repeated mechanical loading. At the molecular level, nanoconfinement provides an additional mechanism for improving mechanical strength and adaptability. Inspired by the two-phase nanostructure of spider silk, Shi et al. fabricated hydrogel microfibers in which hydrogen-bonded nanodomains were embedded within a ductile ionically complexed matrix [30]. This nanoconfined architecture facilitated energy dissipation while preserving network integrity, leading to hydrogels with enhanced mechanical robustness, self-healing capability, damping performance, and humidity-responsive supercontraction.

These studies demonstrate that controlling load transfer and energy dissipation across molecular, fibrous, and hierarchical structural scales is central to balancing strength, toughness, and fatigue resistance in hydrogel systems. These mechanically adaptive architectures broaden the use of robust hydrogels in load-bearing bioelectronics, soft robotics, wearable electronics, and protective biomedical devices.

Despite the high mechanical performance achieved by these biomimetic strategies, several practical limitations must be acknowledged. Prolonged cyclic loading can cause progressive network damage and conductive-pathway disruption, particularly in systems that rely heavily on sacrificial bonds for energy dissipation. In bioelectronic applications, prolonged deformation can also induce signal drift through interfacial water redistribution, polymer-chain relaxation, and gradual changes in conductive pathways. Viscoelastic hysteresis remains another limitation because irreversible or time-dependent deformation can complicate quantitative strain measurement and reduce signal fidelity during dynamic operation. Batch-to-batch reproducibility remains challenging for structurally complex systems, particularly those involving directional freeze-casting or flow-induced alignment, where minor processing variations can significantly affect the final network architecture. These limitations should be systematically characterized and reported alongside performance metrics to provide a balanced assessment of each strategy’s practical viability.

### 3.3. Biomimetic Strategies for Adhesion Properties

Achieving robust adhesion between hydrogels and wet biological tissues remains difficult because interfacial water disrupts molecular interactions and limits intimate contact between adhesive materials and tissue surfaces. Biological systems overcome this limitation through diverse adhesion mechanisms, including suction-assisted attachment, polyphenol-mediated interactions, and catechol-based wet adhesion strategies [40,106,107,108]. Inspired by these biological mechanisms, biomimetic hydrogels have been engineered with microstructured interfaces, dynamic supramolecular networks, and dry-state interfacial crosslinking strategies to achieve durable adhesion under physiological conditions.

**Figure 7:**
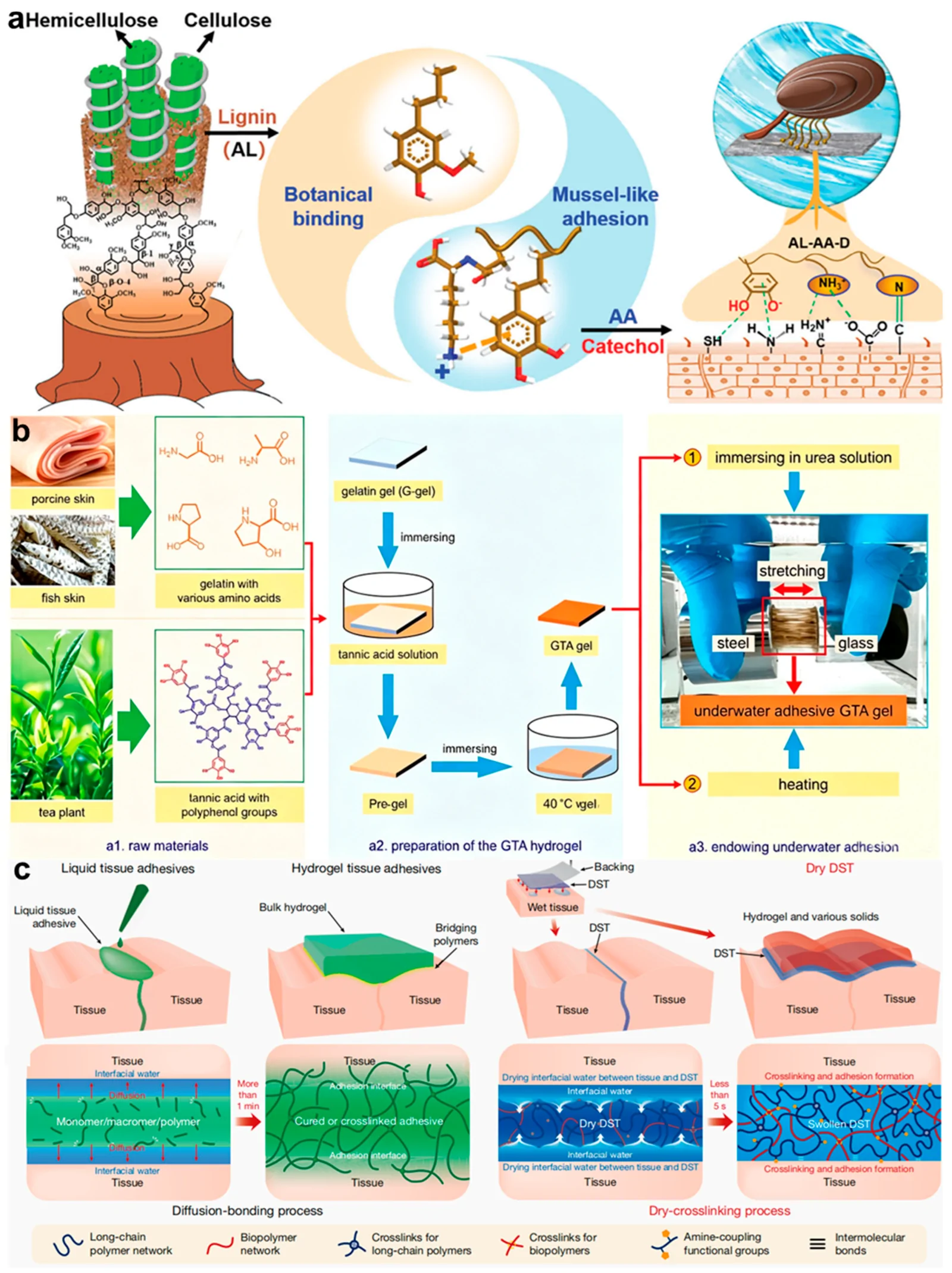
Biomimetic design strategies and functional demonstrations of adhesive hydrogels. (**a**) Schematic illustration of the functional transformation of biomass lignin from botanical binding to bionic adhesion. Amino acid (AA) grafting combining demethylating modification endows alkali lignin with mussel-like adhesion, realizing intramolecular synergy via ion–π/spatial correlation interactions existing in mussel adhesive proteins [38]. (**b**) Schematic illustration of GTA hydrogel fabrication via a stepwise soaking strategy and reversible hydrogen-bond interactions between gelatin and tannic acid, enabling tunable underwater adhesion through urea or thermal treatment [107]. (**c**) Schematic illustration of tissue adhesion mechanisms, comparing diffusion-driven wet adhesives with a dry DST adhesive that achieves adhesion through dehydration-induced contact formation, swelling, and sequential physical and covalent crosslinking [108].

Inspired by the synergistic adhesion of mussel foot proteins, Hu et al. engineered a lignin-based bioadhesive by generating catechol groups through demethylation and subsequently grafting basic amino acids (e.g., lysine) via a Mannich reaction (Figure 7a) [38]. This molecular engineering strategy resulted in a 5.3-fold enhancement in skin adhesion compared with pristine lignin. The enhanced adhesion was attributed to multiple reversible interactions, including hydrogen bonding, cation–π interactions, and Schiff base formation, which were further supported by molecular dynamics simulations. When incorporated into organohydrogel electrodes for wearable bioelectronics, the adhesive layer provided strong tissue coupling, high interfacial toughness (464 J m^−2^), reduced skin–electrode impedance, and improved ECG/EMG signal quality. Dynamic supramolecular interactions provide an alternative molecular strategy for achieving broad-spectrum adhesion. Mimicking polyphenol-mediated bioadhesion, Yuan et al. developed a gelatin–tannic acid (GTA) hydrogel through stepwise assembly without additional chemical crosslinking agents [107]. Extensive hydrogen-bonding interactions simultaneously reinforced the hydrogel network and generated abundant adhesive sites, enabling robust adhesion across diverse substrates and liquid environments while maintaining excellent environmental stability (Figure 7b). Interfacial chemical regulation further improves rapid tissue bonding. Inspired by mussel adhesion, Yuk et al. developed a dry double-sided adhesive tape by integrating gelatin or chitosan with poly(acrylic acid) (PAA) containing N-hydroxysuccinimide (NHS) esters (Figure 7c) [108]. Rapid absorption of interfacial water established intimate tissue contact, followed by hydrogen bonding and covalent coupling between NHS esters and tissue amines, enabling rapid and durable adhesion for tissue sealing and implantable bioelectronic devices.

Recent advances indicate that robust wet adhesion can be achieved through the coordinated regulation of interfacial interactions, structural features, and molecular chemistry. These biomimetic strategies have expanded the application scope of hydrogel adhesives from wearable bioelectronics to implantable devices, tissue repair, and regenerative medicine.

## 4. Applications in Bioelectronics and HMIs

Recent advances in biomimetic hydrogel design have expanded their use from bio-inspired materials to functional platforms for bioelectronics and HMIs [73,109,110,111,112,113]. Owing to their tissue-like mechanics, efficient bioelectrical coupling, and adaptive interfaces, these hydrogels enable stable signal acquisition and transmission between electronic devices and biological tissues [44,74,114,115,116]. These advantages have enabled biomimetic hydrogels to support diverse applications, including electrophysiological monitoring, biochemical sensing, gesture recognition, and robotic control. This section highlights representative applications of biomimetic hydrogels and discusses how bioinspired designs translate material-level properties into mechanical adaptation, interfacial stability, and signal transduction at the device level. Because performance metrics are strongly influenced by testing protocols and operating conditions, direct comparison among studies requires careful consideration of methodological differences. For example, sensitivity measurements can be conducted at substantially different strain rates, while operating temperatures can vary from room temperature to physiological conditions, both of which may substantially influence the measured responses. Similarly, durability is evaluated over different numbers of loading cycles, and adhesion performance is assessed using different methods, substrates, and contact conditions. For electrophysiological monitoring, reported signal-to-noise ratios are also influenced by electrode–skin contact, skin preparation, and signal acquisition parameters. These variations in mechanical, environmental, interfacial, and electrical testing conditions limit direct cross-study comparisons because the measured performance reflects not only material properties but also the specific testing configuration and operating environment. Establishing standardized evaluation protocols and reporting key testing parameters would therefore facilitate more reliable benchmarking and provide a more meaningful basis for assessing the practical performance of biomimetic hydrogel biointerfaces.

### 4.1. Electrophysiological Signal Monitoring

Accurate electrophysiological signal acquisition requires bioelectronic interfaces that can preserve intimate electrical coupling with tissues under mechanical deformation, perspiration, and dynamic physiological conditions [52,54,117,118,119,120]. Although hydrogel electrodes offer inherent softness and ionic conductivity, maintaining stable adhesion, signal quality, and environmental adaptability over extended operation remains challenging. Recent biomimetic strategies, including directional fluid transport, piezoionic signal conversion, and molecular-level interfacial regulation, offer effective approaches for improving the reliability of electrophysiological interfaces [121,122,123,124].

**Figure 8:**
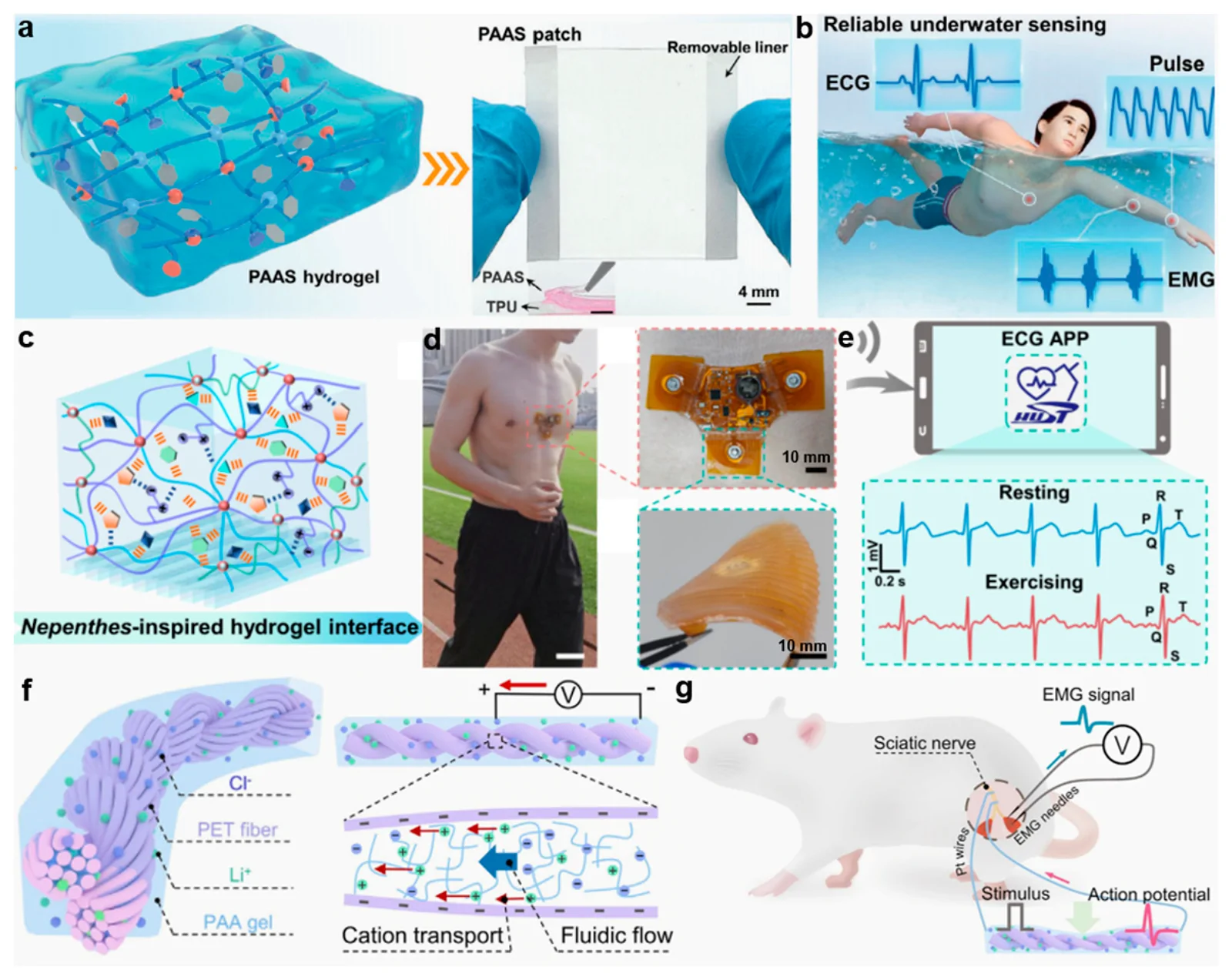
Applications of hydrogels in electrophysiological signal monitoring. (**a**) Schematic illustration of the molecular design and fabrication of the novel hydrogel adhesive (PAAS) hydrogel bioadhesive with asymmetric adhesion enabled by a TPU backing layer [58]. (**b**) Schematic illustration of underwater physiological signal monitoring enabled by the PAAS hydrogel, including pulse, ECG, and EMG recording [58]. (**c**) Schematic illustration of the dual-network architecture of the hydrogel, consisting of a chemically crosslinked poly(acrylic acid)/poly(sulfobetaine methacrylate) network and a physically crosslinked PVA network [59]. (**d**) Photographs demonstrating robust skin–device coupling during exercise and conformal integration of hydrogel electrodes under mechanical deformation [59]. (**e**) Representative ECG signals recorded at rest and during exercise enabled by a conformal hydrogel–skin interface [59]. (**f**) Schematic illustration of a biomimetic artificial nerve featuring oriented microfibers for regulating force-induced ion transport in ionic hydrogels [125]. (**g**) Schematic illustration of a piezoionic artificial nerve for self-powered peripheral nerve stimulation and EMG signal recording [125].

At the molecular level, interfacial engineering can further enhance the stability of hydrogel electrodes under challenging environments. Shen et al. developed a swelling-resistant zwitterionic hydrogel through the integration of hydrophobic interactions and electrostatic regulation (Figure 8a) [58]. The resulting network balanced swelling resistance and interfacial adhesion, enabling reliable electrophysiological recording during prolonged underwater operation (Figure 8b). Furthermore, the hydrogel enabled physiological signal acquisition in large-animal models, demonstrating its suitability for long-term implantable bioelectronic applications. Inspired by the directional liquid transport mechanism of Nepenthes, Yang et al. developed a biomimetic hydrogel electrode incorporating asymmetric sweat-wicking microstructures to regulate interfacial moisture migration (Figure 8c,d) [59]. The directional fluid transport architecture reduced sweat accumulation at the electrode–skin interface, while tannic acid-mediated interactions enhanced wet adhesion and preserved electrical coupling under both dry and perspiring conditions. As a result, the electrode enabled continuous ECG and EMG monitoring during prolonged motion, demonstrating the potential of bioinspired fluid regulation for wearable electrophysiological sensing (Figure 8e). Beyond conventional signal recording, piezoionic signal conversion provides an alternative strategy for constructing biointegrated neural interfaces. Inspired by biological mechanotransduction, Dai et al. developed a self-powered piezoionic artificial nerve by incorporating aligned poly(ethylene terephthalate) (PET) microfibers into a PAA/LiCl hydrogel (Figure 8f) [125]. The anisotropic structure facilitated directional ion migration in response to mechanical stimuli, generating ionic signals that directly triggered peripheral nerve activation without external power sources (Figure 8g). This study demonstrates the capability of biomimetic ionic architectures for interfacing artificial sensing systems with neural tissues. Recent studies demonstrate that regulating hydrogel interfaces through fluid management, ionic signal transduction, and molecular engineering can improve signal fidelity, environmental tolerance, and device durability. These advances establish biomimetic hydrogels as promising platforms for continuous health monitoring, neural interfaces, and integrated wearable bioelectronic systems.

### 4.2. Biochemical Signal Monitoring

Continuous biochemical monitoring enables real-time assessment of physiological states and disease progression but remains limited by invasive sampling procedures, insufficient biofluid availability, and restricted biomarker transport across biointerfaces [63,126,127,128,129]. Biomimetic hydrogels address these limitations by providing hydrated microenvironments, regulating biomolecular interactions, and facilitating efficient analyte transport and signal transduction through bioinspired architectures [130,131,132,133]. Recent advances have integrated ionic–electronic interfaces, flexible optical sensing architectures, and hierarchical conductive networks to couple biomarker transport, enrichment, and signal transduction within wearable sensing platforms.

**Figure 9:**
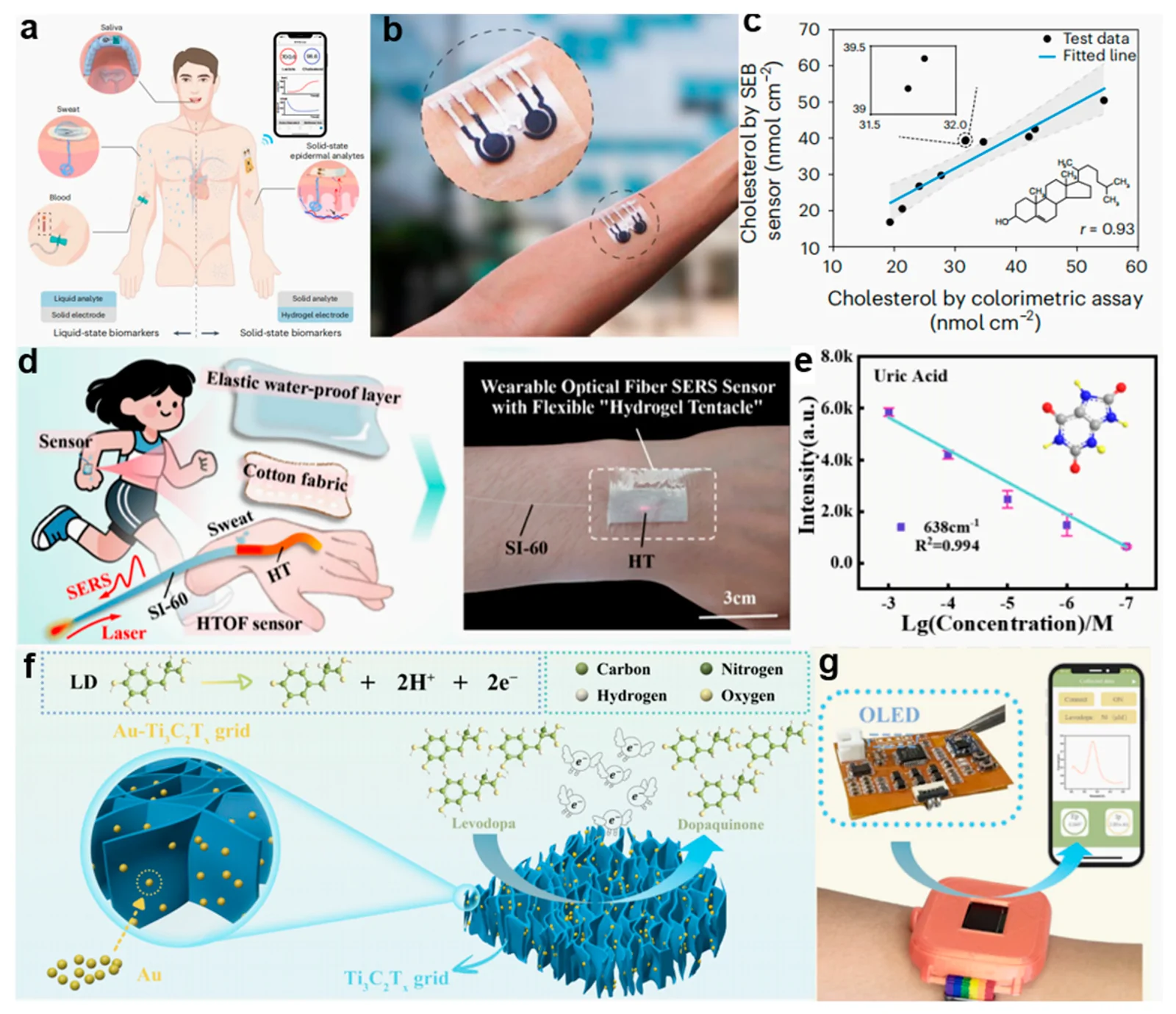
Applications of hydrogels in biochemical signal monitoring. (**a**) Schematic illustration of biofluids (e.g., saliva, sweat, and blood) and SEBs from different body sites, together with corresponding sensor placement strategies for biochemical monitoring [134]. (**b**) Photograph of an SEB sensor conformally attached to human skin [134]. (**c**) Performance evaluation of the SEB sensor for in vivo cholesterol monitoring, demonstrating a strong correlation with standard clinical assays; inset shows an enlarged view of clustered data points [134]. (**d**) Schematic illustration of the sensor packaging architecture, wearable configuration, and mechanical characteristics for epidermal biochemical sensing [63]. (**e**) Correlation between Raman intensity at 638 cm^−1^ and uric acid concentration for biochemical analysis [63]. (**f**) Schematic illustration of the sensing mechanism of a Au-Ti_3_C_2_T_x_grid for electrochemical levodopa detection, involving electrocatalytic oxidation, analyte diffusion through porous structures, and accelerated electron transport within conductive networks [127]. (**g**) Photographs of the wearable levodopa sensor, flexible printed circuit board, and smartphone interface [127].

Biomimetic ionic–electronic interfaces support epidermal biochemical sensing by integrating biomarker transport and electrochemical conversion within a unified architecture. Arwani et al. developed a bilayer hydrogel sensor consisting of an ionic hydrogel layer that maintained a hydrated environment and facilitated biomarker transport, together with an enzyme-functionalized electronic layer for electrochemical signal conversion (Figure 9a) [134]. This integrated design enabled direct detection of solid-state epidermal biomarkers (SEBs) without external biofluid extraction, while mitigating signal fluctuations induced by mechanical motion, demonstrating an effective approach for noninvasive biochemical analysis (Figure 9b,c). Integrating biomarker enrichment with optical signal amplification can further improve the sensitivity of hydrogel-based sensing platforms. Shi et al. developed a flexible hydrogel optical fiber sensor that integrated biomarker collection and surface-enhanced Raman scattering (SERS) detection within a single device (Figure 9d) [63]. The porous Ag nanoparticle–hydrogel network promoted analyte enrichment and enhanced optical interactions, enabling sensitive and reliable detection of multiple sweat biomarkers (Figure 9e). Together with machine-learning-assisted spectral interpretation, the platform achieved accurate biochemical identification, highlighting the potential of hydrogel-enabled optical sensing for wearable health monitoring. Hierarchical conductive architectures offer another route for improving electrochemical biosensing performance. Wei et al. constructed vertically aligned MXene frameworks through electric-field-assisted assembly to facilitate ion transport and increase accessible electroactive sites (Figure 9f) [127]. Integrated with microfluidic sweat sampling and wireless signal transmission, the resulting system enabled continuous levodopa monitoring, illustrating how hierarchical conductive architectures can be coupled with fluidic and wireless components for continuous wearable biochemical sensing.

Recent studies indicate that regulating molecular transport, biomarker enrichment, and interfacial signal conversion is critical for improving the sensitivity, reliability, and practical usability of hydrogel-based biochemical sensors. These developments establish biomimetic hydrogels as multifunctional interfaces for continuous biochemical monitoring, personalized healthcare, and wearable diagnostic systems.

### 4.3. Gesture Recognition

Wearable gesture recognition systems enable intuitive human–machine interaction by converting physiological and mechanical signals into digital commands [22,53,135,136]. However, accurate and reliable gesture decoding remains challenging because of unstable skin–device interfaces, motion-induced signal interference, and insufficient information provided by single sensing modalities. Owing to their tissue-like softness, adaptive interfaces, and tunable electromechanical properties, biomimetic hydrogels have emerged as promising platforms for wearable gesture recognition systems. Recent advances have emphasized the integration of multimodal sensing architectures with signal processing algorithms to enhance decoding accuracy, robustness, and real-time response.

**Figure 10:**
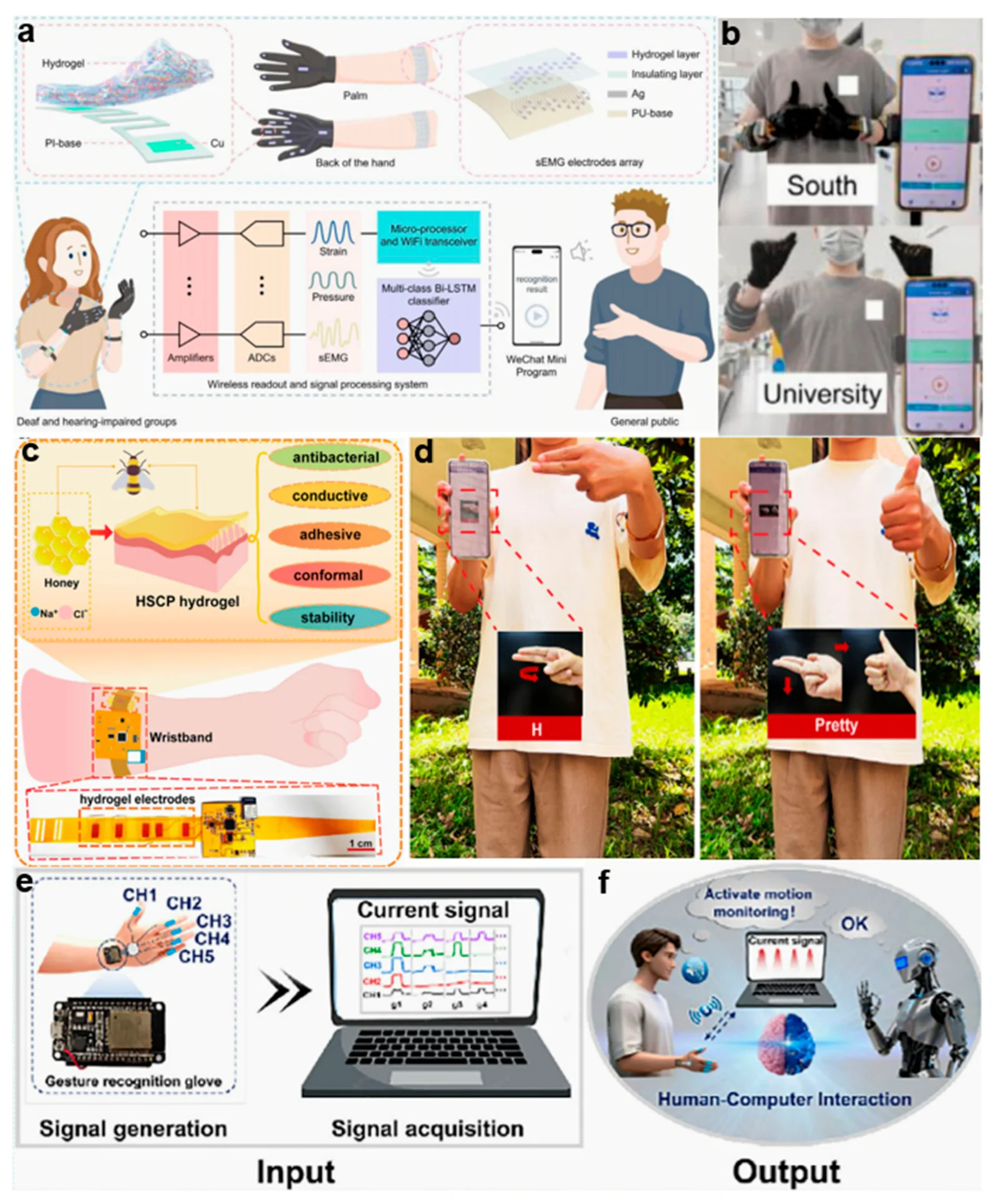
Applications of hydrogels in gesture recognition. (**a**) Schematic illustration of a sign-language recognition system based on tunable ion-conductive hydrogels, integrating strain, pressure, and sEMG sensor arrays for multimodal hand gesture sensing [22]. (**b**) Demonstration of real-time sign-language recognition and translation of “South” and “University” into text and speech through a mobile application interface [22]. (**c**) Schematic illustration of an HMIs based on Honey-NaCl-PAM hydrogel electrodes with integrated self-adhesion, mechanical compliance, stability, conductivity, stretchability, and antibacterial properties, along with a photograph of the wearable wristband device [135]. (**d**) Demonstration of real-time, network-free sign-language translation of “H” and “Pretty” enabled by a mobile HMI system [135]. (**e**) Schematic illustration of a PIVA-Zr^4+^ hydrogel-based gesture-sensing glove integrated with deep learning algorithms for high-precision gesture recognition and human–computer interaction [53]. (**f**) Real time gesture recognition results based on neural network signal analysis.

Multimodal electrophysiological sensing can improve gesture recognition by providing complementary physiological information. Xiao et al. developed a wearable hydrogel wristband by integrating honey–NaCl-modified hydrogel electrodes with surface electromyography (sEMG) sensors and inertial measurement units (Figure 10c) [135]. The bioinspired hydrogel improved skin adhesion and ionic conductivity, enabling stable electrophysiological signal acquisition during continuous movement. Combined with lightweight machine-learning algorithms, the system achieved accurate gesture classification and was further demonstrated for sign language translation, robotic control, and rehabilitation assistance (Figure 10d). Incorporating complementary physiological and mechanical signals can further improve motion decoding accuracy. Hu et al. developed a printable ion-conductive hydrogel with independently tunable mechanical and electrical properties for multimodal gesture sensing (Figure 10a) [22]. The wearable sensing system integrating strain, pressure, and sEMG channels enabled accurate capture of complex hand movements, while multimodal data fusion improved recognition performance compared with single-modality sensing (Figure 10b). This work highlights the importance of combining diverse sensing modalities for reliable interpretation of human motion. Mechanically robust conductive hydrogels provide additional opportunities for long-term wearable interfaces. Zhao et al. fabricated hierarchically crosslinked ion-conductive hydrogels by integrating dynamic covalent bonds, metal coordination, hydrogen bonding, and crystalline domains [53]. The multilevel network architecture simultaneously improved mechanical durability and electrical signal stability, enabling reliable strain sensing under repeated deformation. Coupled with neural-network-based signal analysis, the hydrogel system achieved real-time recognition of diverse hand gestures, emphasizing the role of mechanically adaptive materials in maintaining sensing performance during prolonged use (Figure 10e,f).

Through the integration of adhesive biointerfaces, multimodal signal acquisition, and mechanically resilient conductive networks, biomimetic hydrogels have expanded the capabilities of wearable gesture recognition systems. These systems enable more accurate and robust decoding of human motions, supporting applications in sign language communication, robotic interaction, and personalized rehabilitation.

### 4.4. Robot Control

Hydrogel-based wearable interfaces facilitate intuitive human–robot interaction by integrating tissue-like mechanical compliance, adaptive biointerfaces, and multimodal sensing capabilities [61,62]. However, precise and reliable robotic control requires accurate human intention recognition, real-time perception, and effective feedback communication between users and machines. Inspired by biological sensing and feedback mechanisms, biomimetic hydrogel platforms have integrated self-powered sensing, iontronic tactile perception, and multimodal physiological signal acquisition to support more responsive and adaptive human–robot interaction.

**Figure 11:**
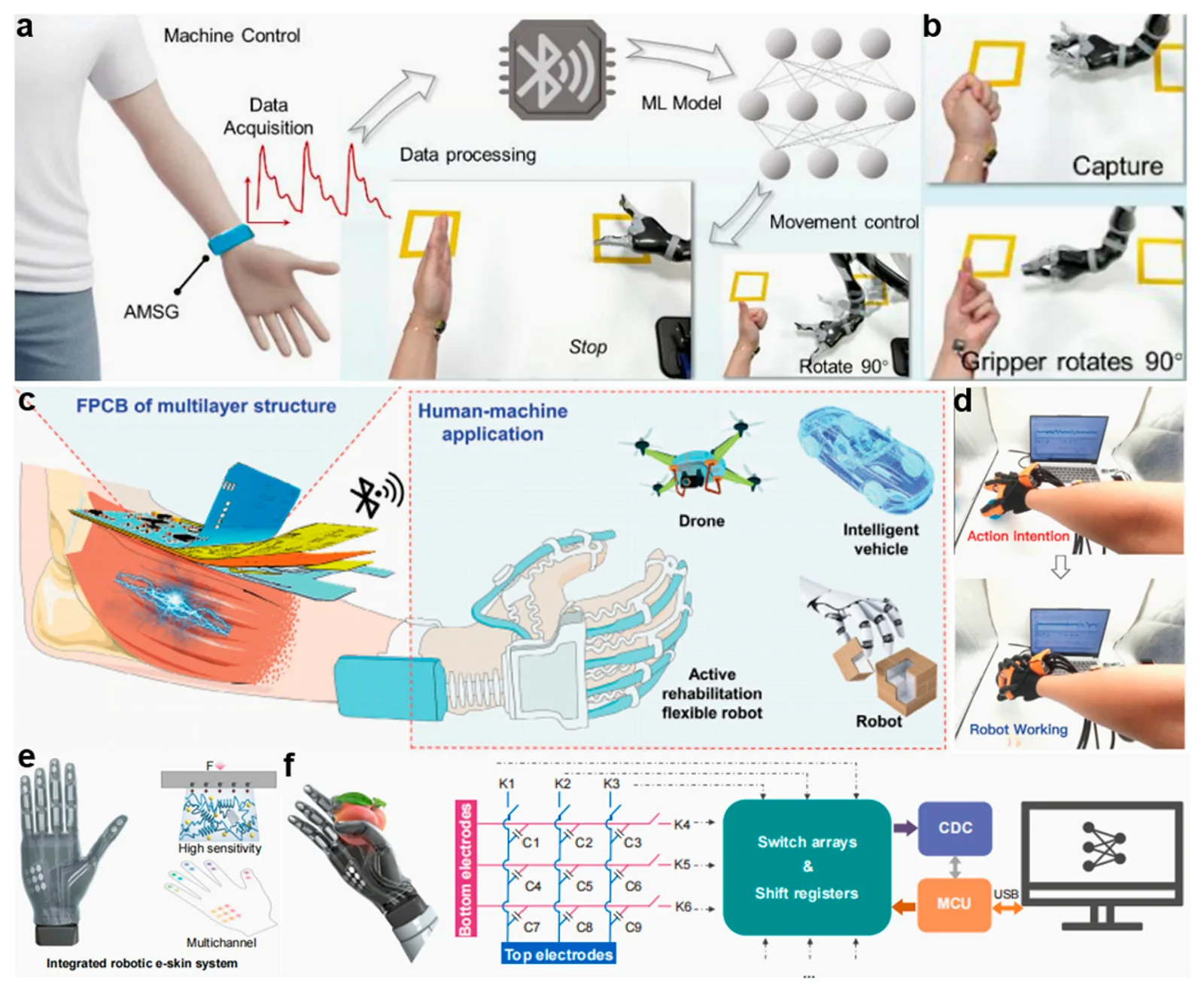
Applications of hydrogels in robot control. (**a**) Schematic illustration of a self-powered active multimodal signal generator (AMSG) wristband-based HMI for wireless gesture recognition and robotic arm control [62]. (**b**) Demonstration of robotic arm control through different hand gestures [62]. (**c**) Schematic illustration of a hydrogel-based wearable human–machine interface integrating EMG and pressure sensing for AI-assisted robotic control in rehabilitation [61]. (**d**) Photographs of a rehabilitation glove enabling intention-driven hand movement assistance [61]. (**e**) Schematic illustration of a multichannel pressure feedback system for precise and non-destructive robotic manipulation [60]. (**f**) Demonstration of an integrated tactile sensing system for adaptive robotic grasping with real-time multichannel pressure feedback [60].

Self-powered multimodal sensing provides a route toward autonomous wearable interfaces for human–robot interaction. Bai et al. developed a self-powered hydrogel electronic skin that integrated thermogalvanic, piezoionic, and diffusion-driven ionic mechanisms within a PVA hydrogel network [62]. The bioinspired system simultaneously generated pressure and thermal signals without external power sources, enabling gesture recognition for robotic arm control. By incorporating haptic feedback, the platform further supported bidirectional communication between users and robotic systems, offering enhanced capabilities for dexterous teleoperation (Figure 11a,b). Iontronic tactile perception provides robots with sensitive tactile information for environmental sensing and physical interaction. Chen et al. developed a gelatin/sodium lactate/montmorillonite iontronic sensor that combined combining ionic crosslinking with bioinspired microstructured surfaces [60].The structured hydrogel exhibited high pressure sensitivity over a broad detection range and was integrated into a robotic hand for multichannel tactile sensing. The resulting system enabled accurate object manipulation and tactile information acquisition, demonstrating the potential of hydrogel-based interfaces for adaptive robotic perception (Figure 11c,d). Beyond external sensing, multimodal physiological signal fusion improves the reliability of human intention decoding. Wang et al. developed a wearable HMI integrating hydrogel-based EMG electrodes with flexible force myography sensors [61]. The combination of complementary physiological signals improved the robustness of gesture recognition compared with single-modality sensing. The system enabled intuitive control of rehabilitation gloves, robotic manipulators, unmanned aerial vehicles, and intelligent vehicles, while allowing wireless transmission of physiological data for remote monitoring and control (Figure 11e,f).

By integrating self-powered sensing, tactile perception, and multimodal physiological decoding, biomimetic hydrogels have broadened the functionality of wearable interfaces for human–robot interaction. Combining compliant biointerfaces with closed-loop feedback and wireless communication enables more adaptive robotic systems for applications in rehabilitation, assistive robotics, teleoperation, and intelligent manufacturing.

## 5. Conclusions and Future Perspectives

Biomimetic hydrogels have emerged as promising biointerface materials for mitigating the mechanical, electrical, and interfacial mismatch between conventional electronics and biological tissues. By incorporating principles derived from biological structures and functions, biomimetic design strategies have enabled hydrogel systems with tissue-matched mechanical compliance, efficient ionic/electronic conduction, robust bioadhesion, and multifunctional sensing functions. These advances have extended the applications of biomimetic hydrogels from electrophysiological monitoring and biochemical sensing to HMIs and robotic systems, demonstrating their versatility as adaptive interfaces between biological tissues and electronic devices.

Despite substantial advances, several challenges remain before biomimetic hydrogels can progress from laboratory demonstrations to practical biomedical applications. A major challenge is the simultaneous optimization of electrical conductivity, mechanical durability, interfacial stability, and long-term biocompatibility, because improving one property often compromises others. Several specific bottlenecks can be identified from the reported literature. First, the trade-off between electrical conductivity and stretchability remains difficult to overcome, with few systems simultaneously achieving high conductivity, high stretchability, and stable performance during prolonged cyclic loading. Second, the long-term stability of hydrogel–tissue interfaces under dynamic physiological conditions, including mechanical deformation, fluid exposure, and immune responses, remains insufficiently validated, particularly for chronic implantation. Third, the lack of standardized testing protocols under realistic operating conditions, such as controlled strain rates, temperature variations, and biofouling environments, limits reliable cross-study comparison of reported performance. Fourth, the integration of soft hydrogel biointerfaces with relatively rigid electronic components remains susceptible to mechanical delamination, interfacial degradation, and contact-resistance drift during repeated deformation. Addressing these material, interface, evaluation, and integration bottlenecks is essential to translating biomimetic hydrogel platforms from proof-of-concept demonstrations into reliable bioelectronic systems.

For implantable systems in particular, long-term operation under physiological conditions requires improved resistance to biofouling, enhanced structural stability, and systematic in vivo evaluation, particularly for implantable bioelectronic systems. The long-term in vivo efficacy of implantable bioelectronics is strongly influenced by progressive material degradation and biofouling. In protein- and polysaccharide-based hydrogels, enzymes such as matrix metalloproteinases and hyaluronidases can promote enzymatic degradation of polymer networks, depending on their chemical composition, thereby compromising mechanical integrity. This structural erosion is exacerbated by reactive oxygen species at the tissue–implant interface, which drive oxidative chain scission and chemical degradation of susceptible polymer linkages. Concurrently, macrophage and fibroblast activity orchestrates both material resorption and fibrotic encapsulation. To mitigate these interfacial fouling processes, zwitterionic surface functionalization is widely employed; its robust hydration layer can reduce nonspecific protein adsorption and cell adhesion. Similarly, PEGylation and biomimetic coatings can reduce fibrotic encapsulation, helping to preserve the bioelectronic interface. Although frequently overlooked, dehydration can substantially compromise hydrogel performance. Under ambient conditions, unprotected hydrogels can undergo substantial water loss, leading to volume shrinkage, mechanical stiffening, and sensor degradation. To mitigate dehydration, humectants such as glycerol, zwitterionic components, and hygroscopic organic solvents can be introduced to form organohydrogels, thereby extending ambient storage stability. Ultimately, clinical and commercial translation will require standardized accelerated-aging protocols, including controlled temperature and humidity conditions, to rigorously assess long-term storage stability.

Future development of biomimetic hydrogels will require not only the reconstruction of biological structures but also the incorporation of adaptive and responsive functions inspired by the dynamic behaviors of living systems. Achieving this goal will require interdisciplinary efforts spanning materials science, bioengineering, electronics, and biomedical engineering to overcome current limitations. Such efforts will be critical for translating biomimetic hydrogels into reliable wearable, implantable, and human–machine interactive bioelectronic systems.

## Figures and Tables

**Table 1:** Comparative summary of polymer-based, carbon–polymer composite, and metal–polymer composite hydrogels for bioelectronics.

Category	Conductivity	Mechanical Properties	Adhesion	Advantages	Limitations	Typical Applications
Polymer-based	0.01–10 S/m	Tunable compliance (10–100 kPa)	Moderate (5–50 kPa)	Excellent biocompatibility; intrinsic ionic conductivity	Low conductivity; dehydration susceptibility	Wearable ECG/EMG; biochemical sensing
Carbon-polymer	1–100 S/m	Enhanced toughness (50–950 kPa)	Moderate to high (20–80 kPa)	High conductivity; tunable network	CNT/graphene cytotoxicity concerns; batch variability	Motion recognition; neural interfaces
Metal-polymer	100–10,000+ S/m	Variable (10–900 kPa)	High (50–200 kPa)	Highest conductivity; stretchable conductors	Filler delamination; oxidation; high cost; limited biodegradability	Stretchable electronics; implantable electrodes

**Table 2:** Comparison of key performance parameters of representative biomimetic hydrogel systems for bioelectronics.

Material System	Conductivity (S m^−1^)	Stretchability (%)	Adhesion (kPa)	Durability (Cycles)	Application
PEDOT:PSS/PVA (polymer)	20	300	N/A	5000	Wearable sensing
Zwitterionic PCBMA (polymer)	0.5	500	N/A	10,000	Silent-speech recognition
CNT/MXene/PAAm (carbon)	8.5	350	25	3000	Motion recognition
CNT/Gelatin (carbon)	1.2	200	45	5000	Neural repair
LM/Ag/PVA (metal)	1000+	400	180	10,000	Stretchable electronics
LM-DN hydrogel (metal)	500	600	N/A	20,000	Strain sensing
AgNW/PNIPAM (metal)	5500	150	N/A	5000	Self-healing electronics
Gelatin-Tannic acid (adhesive)	N/A	250	85	8000	Underwater adhesion
Lignin-Catechol (adhesive)	0.1	400	120	5000	ECG/EMG electrodes
PVA/LiTFSI (piezoionic)	0.3	500	N/A	10,000	Artificial nerve

## Data Availability

No new data were created or analyzed in this study. Data sharing is not applicable to this article.

## References

[1] Jin S.B., Choi H., Seong D., You C.-L., Kang J.-S., Rho S., Lee W.B., Son D., Shin M. (2023). Injectable tissue prosthesis for instantaneous closed-loop rehabilitation. Nature.

[2] Zhang B.B., Li J.Y., Zhou J.K., Chow L., Zhao G., Huang Y., Ma Z., Zhang Q., Yang Y., Yiu C.K. (2024). A three-dimensional liquid diode for soft, integrated permeable electronics. Nature.

[3] Sun Y., Tian G., Deng W., Yang W. (2026). Ionic Hydrogel Sensors toward Next-Gen Personalized Healthcare. Adv. Mater..

[4] Li Z., Ge R., Zhao Z., Xiao H., Du C., Lai Y., Wang L. (2026). From Bio-Interface Materials to Neural Integration: The Next-Generation Brain-Machine Interfaces Powered by Hydrogels. Adv. Mater..

[5] Ye Y.S., Jo Y.J., Oh J.Y., Jeong J.Y., Guo L., Kim T.I. (2026). The Role of Ionic Liquids at the Biological Interfaces in Bioelectronics. Adv. Sci..

[6] Xia M., Liu J., Kim B.J., Gao Y., Zhou Y., Zhang Y., Cao D., Zhao S., Li Y., Ahn J.H. (2024). Kirigami-Structured, Low-Impedance, and Skin-Conformal Electronics for Long-Term Biopotential Monitoring and Human-Machine Interfaces. Adv. Sci..

[7] Li Y., Tan S., Zhang X., Li Z., Cai J., Liu Y. (2025). Design Strategies and Emerging Applications of Conductive Hydrogels in Wearable Sensing. Gels.

[8] Na W., Xu C., An L., Ou C., Gao F., Zhu G., Zhang Y. (2024). Alkali Ion-Accelerated Gelation of MXene-Based Conductive Hydrogel for Flexible Sensing and Machine Learning-Assisted Recognition. Gels.

[9] Han Q.Q., Zhang C., Guo T.M., Tian Y., Song W., Lei J., Li Q., Wang A., Zhang M., Bai S. (2023). Hydrogel Nanoarchitectonics of a Flexible and Self-Adhesive Electrode for Long-Term Wireless Electroencephalogram Recording and High-Accuracy Sustained Attention Evaluation. Adv. Mater..

[10] Tian G.W., Yang D., Liang C.Y., Liu Y., Chen J., Zhao Q., Tang S., Huang J., Xu P., Liu Z. (2023). A Nonswelling Hydrogel with Regenerable High Wet Tissue Adhesion for Bioelectronics. Adv. Mater..

[11] Wang Z.W., Wei H., Huang Y.J., Wei Y., Chen J. (2023). Naturally sourced hydrogels: Emerging fundamental materials for next-generation healthcare sensing. Chem. Soc. Rev..

[12] Mariello M., Rosenthal J.D., Cecchetti F., Gao M., Skrivervik A.K., Leterrier Y., Lacour S.P. (2024). Wireless, battery-free, and real-time monitoring of water permeation across thin-film encapsulation. Nat. Commun..

[13] Nan K.W., Wong K., Li D.F., Ying B., McRae J.C., Feig V.R., Wang S., Du N., Liang Y., Mao Q. (2024). An ingestible, battery-free, tissue-adhering robotic interface for non-invasive and chronic electrostimulation of the gut. Nat. Commun..

[14] Li J., Zhang Y., Song W., Jin Z., Lan T., Shi Q., Xie Y. (2025). Atomic-Layer-Grown Pt on Textile Boosts Adsorption and Sensitivity of MXene Gel Inks for Wearable Electronics. Gels.

[15] Chong J., Sung C., Nam K.S., Kang T., Kim H., Lee H., Park H., Park S., Kang J. (2023). Highly conductive tissue-like hydrogel interface through template-directed assembly. Nat. Commun..

[16] Zhou T., Yuk H., Hu F.Q., Wu J., Tian F., Roh H., Shen Z., Gu G., Xu J., Lu B. (2023). 3D-printable high-performance conducting polymer hydrogel for all-hydrogel bioelectronic interfaces. Nat. Mater..

[17] Lu Y.Y., Yang G., Wang S.Q., Zhang Y., Jian Y., He L., Yu T., Luo H., Kong D., Xianyu Y. (2023). Stretchable graphene-hydrogel interfaces for wearable and implantable bioelectronics. Nat. Electron..

[18] Xia X.J., Liang Q.D., Sun X.G., Yu D., Huang X., Mugo S.M., Chen W., Wang D., Zhang Q. (2022). Intrinsically Electron Conductive, Antibacterial, and Anti-swelling Hydrogels as Implantable Sensors for Bioelectronics. Adv. Funct. Mater..

[19] Leng Z.W., Zhu P.C., Wang X.C., Wang Y., Li P., Huang W., Li B., Jin R., Han N., Wu J. (2023). Sebum-Membrane-Inspired Protein-Based Bioprotonic Hydrogel for Artificial Skin and Human-Machine Merging Interface. Adv. Funct. Mater..

[20] Gao Y., Huo H., Chen Y., Duan X., Li H., Wu W., Qiu C., Ding X., Zhang H., Liu J. (2025). Carbon Dot Bridging Effect Enables the Interweaving of Long- and Short-Chain Polymers for Enhanced Hydrogel Performance. Adv. Funct. Mater..

[21] Yao X., Zhang S., Wei N., Qian L., Coseri S. (2024). Cellulose-Based Conductive Hydrogels for Emerging Intelligent Sensors. Adv. Fiber Mater..

[22] Hu Q., Xiao L., Zhang P., Zhang Q., Liu G., Qin X., Yin Z., Li X., Wang Y., Jiang H. (2026). 3D-Printed Ion-Conductive Hydrogels with Tunable Mechanical-Electrical Properties for Multimodal Sign Language Recognition. Adv. Sci..

[23] Wang S.H., Yu L., Wang S.S., Zhang L., Chen L., Xu X., Song Z., Liu H., Chen C. (2022). Strong, tough, ionic conductive, and freezing-tolerant all-natural hydrogel enabled by cellulose-bentonite coordination interactions. Nat. Commun..

[24] Rahman S., Shon A., Joseph R., Pavlov A., Stefanov A., Namkoong M., Guo H., Bui D., Master R., Sharma A. (2025). Soft, stretchable conductive hydrogels for high-performance electronic implants. Sci. Adv..

[25] Su H.J., Mao L.N., Chen X.Q., Liu P., Pu J., Mao Z., Fujiwara T., Ma Y., Mao X., Li T. (2024). A Complementary Dual-Mode Ion-Electron Conductive Hydrogel Enables Sustained Conductivity for Prolonged Electroencephalogram Recording. Adv. Sci..

[26] Zhang J., Shen S., Lin R.R., Huang J., Pu C., Chen P., Duan Q., You X., Xu C., Yan B. (2023). Highly Stretchable and Biocompatible Wrinkled Nanoclay-Composite Hydrogel with Enhanced Sensing Capability for Precise Detection of Myocardial Infarction. Adv. Mater..

[27] Wu J.Y., Zhang Z.X., Wu Z.Y., Liu D., Yang X., Wang Y., Jia X., Xu X., Jiang P., Wang X. (2022). Strong and Ultra-Tough Supramolecular Hydrogel Enabled by Strain-Induced Microphase Separation. Adv. Funct. Mater..

[28] Chu C.Z., Sun W., Chen S., Jia Y., Ni Y., Wang S., Han Y., Zuo H., Chen H., You Z. (2024). Squid-Inspired Anti-Salt Skin-Like Elastomers with Superhigh Damage Resistance for Aquatic Soft Robots. Adv. Mater..

[29] Xu F.C., Li H., Li Y. (2024). Sea Cucumber-Inspired Polyurethane Demonstrating Record-Breaking Mechanical Properties in Room-Temperature Self-Healing Ionogels. Adv. Mater..

[30] Shi Y.K., Wu B.H., Sun S.T., Wu P. (2023). Aqueous spinning of robust, self-healable, and crack-resistant hydrogel microfibers enabled by hydrogen bond nanoconfinement. Nat. Commun..

[31] Wu S.J., Liu Z., Gong C.H., Li W., Xu S., Wen R., Feng W., Qiu Z., Yan Y. (2024). Spider-silk-inspired strong and tough hydrogel fibers with anti-freezing and water retention properties. Nat. Commun..

[32] Amoli V., Kim J.S., Jee E., Chung Y.S., Kim S.Y., Koo J., Choi H., Kim Y., Kim D.H. (2019). A bioinspired hydrogen bond-triggered ultrasensitive ionic mechanoreceptor skin. Nat. Commun..

[33] Fang Y., Ouyang H., Cheng Y., Zhou Y., Shi L., Sun J., Ho G.W., Wang R. (2025). Ultrasensitive multi-degree-of-freedom piezoionic sensor via synergistic hydrogel-ion interactions. Nat. Commun..

[34] Xu S.J., Yu J.X., Guo H.S., Tian S., Long Y., Yang J., Zhang L. (2023). Force-induced ion generation in zwitterionic hydrogels for a sensitive silent-speech sensor. Nat. Commun..

[35] Bai M., Chen Y.R., Zhu L.Y., Li Y., Ma T., Li Y., Qin M., Wang W., Cao Y., Xue B. (2024). Bioinspired adaptive lipid-integrated bilayer coating for enhancing dynamic water retention in hydrogel-based flexible sensors. Nat. Commun..

[36] Wang H., Wang S., Jiang Y., Han Z., Lin D., Luo Q., Fu T., Zhang H., Yu D., Yi J. (2026). Bioinspired Directional Hydrogel-Based High-Performance Flexible Sensor for Multiple Jumping Pattern Detection in Athletic Training. Adv. Sci..

[37] Shi D., Ji D., Bae J. (2025). Hierarchically structuralized hydrogels with ligament-like mechanical performance. Nat. Commun..

[38] Hu O.D., Lu M.J., Cai M., Liu J., Qiu X., Guo C.F., Zhang C.Y., Qian Y. (2024). Mussel-Bioinspired Lignin Adhesive for Wearable Bioelectrodes. Adv. Mater..

[39] Liao H.G., Hu S., Yang H., Wang L., Tanaka S., Takigawa I., Li W., Fan H., Gong J.P. (2025). Data-driven de novo design of super-adhesive hydrogels. Nature.

[40] Deng T., Gao D.X., Song X.M., Zhou Z., Zhou L., Tao M., Jiang Z., Yang L., Luo L., Zhou A. (2023). A natural biological adhesive from snail mucus for wound repair. Nat. Commun..

[41] Xue B., Gu J., Li L., Yu W., Yin S., Qin M., Jiang Q., Wang W., Cao Y. (2021). Hydrogel tapes for fault-tolerant strong wet adhesion. Nat. Commun..

[42] Wang C., Wang H., Wang B., Miyata H., Wang Y., Nayeem O.G., Kim J.J., Lee S., Yokota T., Onodera H. (2022). On-skin paintable biogel for long-term high-fidelity electroencephalogram recording. Sci. Adv..

[43] Zhang L., Kumar K.S., He H., Cai C.J., He X., Gao H., Yue S., Li C., Seet R.C.-S., Ren H. (2020). Fully organic compliant dry electrodes self-adhesive to skin for long-term motion-robust epidermal biopotential monitoring. Nat. Commun..

[44] Luo J.B., Sun C.Y., Chang B.Y., Jing Y.M., Li K., Li Y., Zhang Q., Wang H., Hou C. (2022). MXene-Enabled Self-Adaptive Hydrogel Interface for Active Electroencephalogram Interactions. ACS Nano.

[45] Yang C.W., Li P.J., Wei C., Prominski A., Ma J., Sun C., Yue J., Cheng Z., Zhang J., Ashwood B. (2024). A bioinspired permeable junction approach for sustainable device microfabrication. Nat. Sustain..

[46] Reis Carneiro M., Lopes T., Silva A.F., Majidi C., Tavakoli M. (2026). Micropatterned Biphasic Printed Electrodes for High-Fidelity on-Skin Bioelectronics. Adv. Funct. Mater..

[47] Li X., Zhou Y., Wei Z., Yin H., Wang P., Li G., Meng C. (2026). High-Performance Hydrogel-Based Temperature Sensor by Integrating Ion Channels and Stabilized Ion Gradients. Adv. Funct. Mater..

[48] Zhu M., Gong D., Ji Z., Yang J., Wang M., Wang Z., Tao S., Wang X., Xu M. (2023). Cellulose-reinforced poly(Ionic Liquids) composite hydrogel for infected wounds therapy and real-time reliable bioelectronic. Chem. Eng. J..

[49] Hu Y., Yang Y., Chu H., Shi R., Li J., Xu G., Huang X., Zhang B., Yiu C.K., Zhao G. (2025). Prolonged monitoring and risk management of hyperuricemia using interference-resistant wearable bioelectronics. Device.

[50] Kanokpaka P., Chang Y.-H., Chang C.-C., Rinawati M., Wang P.-C., Chang L.-Y., Yeh M.-H. (2023). Enabling glucose adaptive self-healing hydrogel based triboelectric biosensor for tracking a human perspiration. Nano Energy.

[51] Liu Y., Yang T., Zhang Y., Qu G., Wei S., Liu Z., Kong T. (2019). Ultrastretchable and Wireless Bioelectronics Based on All-Hydrogel Microfluidics. Adv. Mater..

[52] Zhu P.C., Niu M.J., Liang S.Y., Yang W., Zhang Y., Chen K., Pan Z., Mao Y. (2025). Non-hand-worn, load-free VR hand rehabilitation system assisted by deep learning based on ionic hydrogel. Nano Res..

[53] Zhao W., Wei X., Shi R., Zhao Y., Liu R., Guo J., Li W., Xu A., Lu W., Zhao L. (2026). Engineering Tough and Stretchable Ion-Conductive Hydrogels with Hierarchical Dynamic Bonds for Deep Learning-Assisted Gesture Recognition. Adv. Funct. Mater..

[54] Li X.L., Sun Y., Wang S.L., Tian G., Yang T., Huang L., Ao Y., Lan B., Zhang J., Xu T. (2024). Body temperature-triggered adhesive ionic conductive hydrogels for bioelectrical signal monitoring. Chem. Eng. J..

[55] Yi B., Li T.J., Yang B.G., Chen S.R., Zhao J., Zhao P., Zhang K., Wang Y., Wang Z., Bian L. (2024). Surface hydrophobization of hydrogels via interface dynamics-induced network reconfiguration. Nat. Commun..

[56] Lei W.L., Peng C.W., Chiu S.C., Lu H.E., Wu C.W., Cheng T.Y., Huang W.C. (2023). All Biodisintegratable Hydrogel Biohybrid Neural Interfaces with Synergistic Performances of Microelectrode Array Technologies, Tissue Scaffolding, and Cell Therapy. Adv. Funct. Mater..

[57] Hao X.P., Li C.Y., Zhang C.W., Du M., Ying Z., Zheng Q., Wu Z.L. (2021). Self-Shaping Soft Electronics Based on Patterned Hydrogel with Stencil-Printed Liquid Metal. Adv. Funct. Mater..

[58] Shen K.X., Lv Z.T., Yang Y.X., Wang H., Liu J., Chen Q., Liu Z., Zhang M., Liu J., Cheng Y. (2024). A Wet-Adhesion and Swelling-Resistant Hydrogel for Fast Hemostasis, Accelerated Tissue Injury Healing and Bioelectronics. Adv. Mater..

[59] Yang G.G., Lan Z.G., Gong H.Y., Wen J., Pang B., Qiu Y., Zhang Y., Guo W., Bu T., Xie B. (2024). A Nepenthes-Inspired Hydrogel Hybrid System for Sweat-Wicking Electrophysiological Signal Recording during Exercises. Adv. Funct. Mater..

[60] Chen Y., Wang S., Ye X., Lv C., Wei J., Zhang Y., Ping J., Ying Y., Lan L. (2026). Skin-mimicking biogel-based iontronic sensor with hierarchical bionic coupling for dexterous tactile e-skin. Nat. Commun..

[61] Wang H., Ding Q., Luo Y., Wu Z., Yu J., Chen H., Zhou Y., Zhang H., Tao K., Chen X. (2024). High-Performance Hydrogel Sensors Enabled Multimodal and Accurate Human-Machine Interaction System for Active Rehabilitation. Adv. Mater..

[62] Bai C., Dong X., Liu Q., Zhao M., Yang K., Niu Y., Zhang H., Zhai W. (2026). A self-powered hydrogel electronic skin with decoupled multimodal sensing for closed-loop human-machine interactions. Nat. Commun..

[63] Shi C., Yang X., Liu Z., Yang J., Zhang J., Tian F., Li X., Wang R., Lu N., Li K. (2026). Wearable Optical Fiber SERS Sensor Based on a Flexible “Hydrogel Tentacle” for Sweat Remote and In Situ Detection. ACS Sens..

[64] Song Y., Tay R.Y., Li J., Xu C., Min J., Sani E.S., Kim G., Heng W., Kim I., Gao W. (2023). 3D-Printed epifluidic electronic skin for machinelearning-powered multimodal health surveillance. Sci. Adv..

[65] Zhuang Q.N., Yao K.M., Zhang C., Song X., Zhou J., Zhang Y., Huang Q., Zhou Y., Yu X., Zheng Z. (2024). Permeable, three-dimensional integrated electronic skins with stretchable hybrid liquid metal solders. Nat. Electron..

[66] Wang X., Dong L., Zhang H., Yu R., Pan C., Wang Z.L. (2015). Recent Progress in Electronic Skin. Adv. Sci..

[67] Li G., Huang K.X., Deng J., Guo M., Cai M., Zhang Y., Guo C.F. (2022). Highly Conducting and Stretchable Double-Network Hydrogel for Soft Bioelectronics. Adv. Mater..

[68] Gao Y., Chen J., Han X., Pan Y., Wang P., Wang T., Lu T. (2020). A Universal Strategy for Tough Adhesion of Wet Soft Material. Adv. Funct. Mater..

[69] Eklund A., Ikkala O., Zhang H. (2023). Highly Efficient Switchable Underwater Adhesion in Channeled Hydrogel Networks. Adv. Funct. Mater..

[70] Wu M., Qiao C., Sui P.F., Luo J.L., Li Z., Cao Y., Pei R., Peng X., Zeng H. (2025). Stratum Corneum-Inspired Zwitterionic Hydrogels with Intrinsic Water Retention and Anti-Freezing Properties for Intelligent Flexible Sensors. Adv. Funct. Mater..

[71] Liu Y., Wang C., Xue J.T., Huang G., Zheng S., Zhao K., Huang J., Wang Y., Zhang Y., Yin T. (2022). Body Temperature Enhanced Adhesive, Antibacterial, and Recyclable Ionic Hydrogel for Epidermal Electrophysiological Monitoring. Adv. Healthc. Mater..

[72] Zeng J., Chen H., Dong L.B., Guo X. (2024). Anti-Polyelectrolyte Effect of Zwitterionic Hydrogel Electrolytes Enabling High-Voltage Zinc-Ion Hybrid Capacitors. Adv. Funct. Mater..

[73] Yu D.S., Yi J., Zhu S.H., Tang Y.H., Huang Y.Y., Lin D., Lin Y. (2024). Hofmeister Effect-Assisted Facile One-Pot Fabrication of Double Network Organohydrogels with Exceptional Multi-Functions. Adv. Funct. Mater..

[74] Yao X., Zhang S.F., Qian L.W., Wei N., Nica V., Coseri S., Han F. (2022). Super Stretchable, Self-Healing, Adhesive Ionic Conductive Hydrogels Based on Tailor-Made Ionic Liquid for High-Performance Strain Sensors. Adv. Funct. Mater..

[75] Liu Y., Wang X., Wang J., Li Z., Ao K., Liang G., Liu H., Zhang Q., Pan M., Shou D. (2026). Bioinspired Structural Design Enables Synergistic Toughness and Conductivity in Hydrogels for Advanced Wearable Electronics. Nano-Micro Lett..

[76] Lan L., Ping J., Li H., Wang C., Li G., Song J., Ying Y. (2024). Skin-Inspired All-Natural Biogel for Bioadhesive Interface. Adv. Mater..

[77] Tan Y., Wang Y., Zhang P., Li J., Wang F., Shan L., Guo J., Wang Z., Liu J. (2025). Bioinspired Hierarchical Hydrogels Engineered with Extreme Impact Resistance. Adv. Funct. Mater..

[78] Kim J., Park S., Jeon J., Kang D.-h., Bryan G.M., Broderick T.J., Stone M., Tsukruk V.V. (2025). Robust Skin-Conformal Nano-Electrodes for Sustainable Health and Performance Monitoring. ACS Nano.

[79] Lee S., Ho D.H., Jekal J., Cho S.Y., Choi Y.J., Oh S., Choi Y.Y., Lee T., Jang K.-I., Cho J.H. (2024). Fabric-based lamina emergent MXene-based electrode for electrophysiological monitoring. Nat. Commun..

[80] Xu P.F., Wang S.J., Lin A., Min H.-K., Zhou Z., Dou W., Sun Y., Huang X., Tran H., Liu X. (2023). Conductive and elastic bottlebrush elastomers for ultrasoft electronics. Nat. Commun..

[81] Qiu R.J., Zhang X.Y., Song C., Xu K., Nong H., Li Y., Xing X., Mequanint K., Liu Q., Yuan Q. (2024). E-cardiac patch to sense and repair infarcted myocardium. Nat. Commun..

[82] Das P., Ganguly S., Marvi P.K., Sherazee M., Tang X.S., Srinivasan S., Rajabzadeh A.R. (2024). Carbon Dots Infused 3D Printed Cephalopod Mimetic Bactericidal and Antioxidant Hydrogel for Uniaxial Mechano-Fluorescent Tactile Sensor. Adv. Mater..

[83] Wang S., Wang Z., Yang W., Xu Z., Dai H., He F., Yan S., Shi X. (2024). In Situ-Sprayed Bioinspired Adhesive Conductive Hydrogels for Cavernous Nerve Repair. Adv. Mater..

[84] Jiang M., Han C., Cao Z., Zhao Y., Zhao X., Zhou K., Zhai W., Zheng G., Zhu X., Wan P. (2025). Microcrack-Structured Visualizable Hydrogel Sensor for Machine Learning-Assisted Handwriting Recognition. Adv. Funct. Mater..

[85] Li N., Li Y., Cheng Z., Liu Y., Dai Y., Kang S., Li S., Shan N., Wai S., Ziaja A. (2023). Bioadhesive polymer semiconductors andtransistors for intimate biointerfaces. Science.

[86] Deng J., Yuk H., Wu J.J., Varela C.E., Chen X., Roche E.T., Guo C.F., Zhao X. (2020). Electrical bioadhesive interface for bioelectronics. Nat. Mater..

[87] Zhang X.Y., Li W., Li H.J., Xuan X., Li C., Li M. (2025). Flexible Au-Pt-vertical graphene neural microelectrode for the rapid detection of steady-state and transient dopamine in rats. Biosens. Bioelectron..

[88] Cao C.Y., Hou C.S., Wang X., Lv D., Ai L., Feng Y., Chen P., Wang X., He M., Yao X. (2024). Liquid Metal-Enhanced Highly Adhesive Electrodes for Multifunctional Epidermal Bioelectronics. Adv. Funct. Mater..

[89] Li X., Zhu P.C., Zhang S.C., Wang X., Luo X., Leng Z., Zhou H., Pan Z., Mao Y. (2022). A Self-Supporting, Conductor-Exposing, Stretchable, Ultrathin, and Recyclable Kirigami-Structured Liquid Metal Paper for Multifunctional E-Skin. ACS Nano.

[90] Bao D., Guan F., Ji X., Zhang X., Xu Y., Yang Q., Yao Q., Zhang S., Guo J. (2025). Unidirectionally arranged layered structured hydrogels with high strength, multifunctional integration, and somatosensory actuators. Chem. Eng. J..

[91] Jiao B., Wang W., Feng Y., Zhou D., Wang X., Zhou Y., Wang X., Shang Y., Wang Q. (2026). High-strength liquid metal composite-hydrogel interfaces enable robust stretchable electronics. Nat. Commun..

[92] Cui P., Chen J., Chen R., Chang W., Chen K., Yuan C., Wang X., Wang S. (2026). Liquid Metal-Reinfored Hierarchically Aligned Double-Network Hydrogels: Ultrahigh Crack/Fatigue Resistance and Strain-Responsive Sensing. Small.

[93] Song P., Qin H., Gao H.L., Cong H.P., Yu S.H. (2018). Self-healing and superstretchable conductors from hierarchical nanowire assemblies. Nat. Commun..

[94] Zhao B.C., Yan J.Q., Long F., Qiu W., Meng G., Zeng Z., Huang H., Wang H., Lin N., Liu X.Y. (2023). Bioinspired Conductive Enhanced Polyurethane Ionic Skin as Reliable Multifunctional Sensors. Adv. Sci..

[95] Yu C.J., Shi M.Y., He S.S., Yao M., Sun H., Yue Z., Qiu Y., Liu B., Liang L., Zhao Z. (2023). Chronological adhesive cardiac patch for synchronous mechanophysiological monitoring and electrocoupling therapy. Nat. Commun..

[96] Zhang W., Wu B.H., Sun S.T., Wu P. (2021). Skin-like mechanoresponsive self-healing ionic elastomer from supramolecular zwitterionic network. Nat. Commun..

[97] Li X.N., Tong Z.H., Qiu Y.X., Zheng Z., Zeng S., Zhao D., Yu H. (2025). Balancing mechanical-thermal-electrical properties in cellulose ionogels via crystallization-induced molecular assembly. Nat. Commun..

[98] Han F., Chen S.M., Wang F., Liu M., Li J., Liu H., Yang Y., Zhang H., Liu D., He R. (2025). High-Conductivity, Self-Healing, and Adhesive Ionic Hydrogels for Health Monitoring and Human-Machine Interactions Under Extreme Cold Conditions. Adv. Sci..

[99] Chen B., Yu R., Wang J., Feng Y., Zhang Y., Mao Y., Shan C., Wang X. (2025). Biomaterials-Based Hydrogel with Superior Bio-Mimetic Ionic Conductivity and Tissue-Matching Softness for Bioelectronics. Adv. Funct. Mater..

[100] Akbar T.F., Jimenez-Rodriguez C.A., Biktimirova R., Hermes I., Kurth T., Pham M.D., Tsurkan M., Friedrichs J., Morgan F.L.C., Kleemann H. (2026). Conductive Hydrogels for Exogenous Sensing and Cell Fate Control. Adv. Mater..

[101] Wu K., Lu C., Yuan F., Song B., Lei K., Wang Z., Wang H., Peng L., Qi H., Ji H. (2026). A Protein-Managed Hydrogel Biomimicked by Insect Cuticle Enabling Ultra-Durable Impact Resistance. Adv. Mater..

[102] Xue C., Zhao Y., Liao Y., Zhang H. (2025). Bioinspired Super-Robust Conductive Hydrogels for Machine Learning-Assisted Tactile Perception System. Adv. Mater..

[103] Zhong W., Zhao H., Yao B., Li Z., Zhou S., Wang Z., Geng Y., Sun W., Fu J. (2025). Highly reconfigurable neuronlike conductive networks through nanophase structure engineering. Nat. Commun..

[104] Ma Y.F., Hua M.T., Wu S.W., Du Y.J., Pei X., Zhu X., Zhou F., He X. (2020). Bioinspired high-power-density strong contractilehydrogel by programmable elastic recoil. Sci. Adv..

[105] Feng Y., Wang Y., Wang C., Chen X., Shan L., Yan R., Wang Z., Liu S., Liu J. (2026). Fatigue Resistant Hydrogels Engineered with Twisting Hierarchical Structures. Adv. Mater..

[106] Zheng Z., Chen X., Wang Y., Wen P., Duan Q., Zhang P., Shan L., Ni Z., Feng Y., Xue Y. (2024). Self-Growing Hydrogel Bioadhesives for Chronic Wound Management. Adv. Mater..

[107] Yuan Z., Wang T., Shao C., Yang S., Sun W., Chen Y., Peng Z., Tong Z. (2024). Bioinspired Green Underwater Adhesive Gelatin-Tannic Acid Hydrogel with Wide Range Adjustable Adhesion Strength and Multiple Environmental Adaptability. Adv. Funct. Mater..

[108] Yuk H., Varela C.E., Nabzdyk C.S., Mao X., Padera R.F., Roche E.T., Zhao X. (2019). Dry double-sided tape for adhesion of wet tissues and devices. Nature.

[109] Li H., Cao J., Wan R., Feig V.R., Tringides C.M., Xu J., Yuk H., Lu B. (2024). PEDOTs-Based Conductive Hydrogels: Design, Fabrications, and Applications. Adv. Mater..

[110] Gao G., Yang F., Zhou F., He J., Lu W., Xiao P., Yan H., Pan C., Chen T., Wang Z.L. (2020). Bioinspired Self-Healing Human–Machine Interactive Touch Pad with Pressure-Sensitive Adhesiveness on Targeted Substrates. Adv. Mater..

[111] Cao X., Ye C., Cao L., Shan Y., Ren J., Ling S. (2023). Biomimetic Spun Silk Ionotronic Fibers for Intelligent Discrimination of Motions and Tactile Stimuli. Adv. Mater..

[112] Lu L., Chen X., Zhang Y., Liu J., Liu C., Hu Y., Mao Y. (2026). Bioinspired Ion-Proton Bipathway All-Natural Hydrogel with Enhanced Conductivity for Bioelectronic Interfaces. Adv. Funct. Mater..

[113] Zhu P., Zhang Y., Wu A., Feng Y., Liu Y., Niu M., Han N., Lin Y., Pan Z., Mao Y. (2026). A damping and adhesive hydrogel electrode for continuous high-fidelity dynamic electrophysiological monitoring and human–machine interaction. Nano Res..

[114] He Q.H., Cheng Y., Deng Y.J., Wen F., Lai Y., Li H. (2023). Conductive Hydrogel for Flexible Bioelectronic Device: Current Progress and Future Perspective. Adv. Funct. Mater..

[115] Chen L.R., Chang X.H., Chen J.W., Zhu Y. (2022). Ultrastretchable, Antifreezing, and High-Performance Strain Sensor Based on a Muscle-Inspired Anisotropic Conductive Hydrogel for Human Motion Monitoring and Wireless Transmission. ACS Appl. Mater. Interfaces.

[116] Park J., Kim H.W., Lim S., Yi H., Wu Z., Kwon I.G., Yeo W.H., Song E., Yu K.J. (2024). Conformal Fixation Strategies and Bioadhesives for Soft Bioelectronics. Adv. Funct. Mater..

[117] Zhang Z.M., Yang J.W., Wang H.Y., Wang C.Y., Gu Y.H., Xu Y.H. (2024). A 10-micrometer-thick nanomesh-reinforcedgas-permeable hydrogel skin sensor for long-termelectrophysiological monitoring. Sci. Adv..

[118] Wang Y.L., Wang Z.L., Zhang Y.J., Yang J.W., Zhang Z.M., Zhou P.C., Xu Y.M. (2025). A 2.7-μm-thick robust, permeable, and antifreezinghydrogel electrode for long-term ambulatoryhealth monitoring. Sci. Adv..

[119] Lee S., Kum J., Kim S., Jung H., An S., Choi S.J., Choi J.H., Kim J., Yu K.J., Lee W. (2024). A shape-morphing cortex-adhesive sensor for closed-loop transcranial ultrasound neurostimulation. Nat. Electron..

[120] Wang X., Sun X.T., Gan D.L., Soubrier M., Chiang H.-Y., Yan L., Li Y., Li J., Yu S., Xia Y. (2022). Bioadhesive and conductive hydrogel-integrated brain-machine interfaces for conformal and immune-evasive contact with brain tissue. Matter.

[121] Rodriguez-Rivera G.J., Post A., John M., Buchan S., Bernard D., Razavi M., Cosgriff-Hernandez E. (2024). Injectable hydrogel electrodes as conduction highways to restore native pacing. Nat. Commun..

[122] Yuan X.M., Zhu Z., Xia P.C., Wang Z., Zhao X., Jiang X., Wang T., Gao Q., Xu J., Shan D. (2023). Tough Gelatin Hydrogel for Tissue Engineering. Adv. Sci..

[123] Wang F.C., Xue Y., Chen X.M., Zhang P., Shan L., Duan Q., Xing J., Lan Y., Lu B., Liu J. (2023). 3D Printed Implantable Hydrogel Bioelectronics for Electrophysiological Monitoring and Electrical Modulation. Adv. Funct. Mater..

[124] Lee M., Park J., Choe G., Lee S., Kang B.G., Jun J.H., Shin Y., Kim M.C., Kim Y.S., Ahn Y. (2023). A Conductive and Adhesive Hydrogel Composed of MXene Nanoflakes as a Paintable Cardiac Patch for Infarcted Heart Repair. ACS Nano.

[125] Dai J.Q., Xue Y., Chen X.M., Cao Z.W., Wang L., Zhang J., Zhou Y., Hu Y., Zhou W., Tang W. (2024). Artificial nerve for neuromodulation based on structured piezoionic hydrogel. Device.

[126] Chu H., Park W., Hu Y., Huang Z., Shi R., Li J., Li Z., Wu M., Gao Y., Zhao G. (2025). Assessment of Acute Stress and Chronic Stress for Mental Health Management by a Fully Integrated Wearable Biosystem. ACS Nano.

[127] Wei N., Cheng J., Wang X., Shen B., Chuai Y., Zhang Y. (2026). Vertically Oriented Assembly of a Few-Layer Ti_3_C_2_T_x_ Grid by Electric Field Inducing and Freeze Drying for Wearable Levodopa Sensing in Sweat. ACS Sens..

[128] Zhu Y., Haghniaz R., Hartel M.C., Guan S., Bahari J., Li Z., Baidya A., Cao K., Gao X., Li J. (2023). A Breathable, Passive-Cooling, Non-Inflammatory, and Biodegradable Aerogel Electronic Skin for Wearable Physical-Electrophysiological-Chemical Analysis. Adv. Mater..

[129] Qin Y., Mo J., Liu Y., Zhang S., Wang J., Fu Q., Wang S., Nie S. (2022). Stretchable Triboelectric Self-Powered Sweat Sensor Fabricated from Self-Healing Nanocellulose Hydrogels. Adv. Funct. Mater..

[130] Garcia-Rey S., Gil-Hernandez E., Gunatilake U.B., Basabe-Desmonts L., Benito-Lopez F. (2023). Development of an alginate/TiO_2_-based microfluidic biosystem for chrono-sampling and sensing of glucose in artificial sweat. Sens. Actuators B Chem..

[131] Wang C., Wang Z., Wei W., Zhang Z., Li A.A., Huang G., Li X., Ge S.S., Zhou L., Kong H. (2024). High-precision flexible sweat self-collection sensor for mental stress evaluation. npj Flex. Electron..

[132] Li Q., He C., Wang C., Huang Y., Yu J., Wang C., Li W., Zhang X., Zhang F., Qing G. (2023). Sustainable, Insoluble, and Photonic Cellulose Nanocrystal Patches for Calcium Ion Sensing in Sweat. Small.

[133] He X., Li Z., Huang X., Zhang Q., Zeng Y., Li J., Yiu C.K., Yang Y., Zhou J., Xu G. (2024). Sweat-powered, skin-adhesive multimodal sensor for long-term and real-time sweat monitoring. BMEMat.

[134] Arwani R.T., Tan S.C.L., Sundarapandi A., Goh W.P., Liu Y., Leong F.Y., Yang W., Zheng X.T., Yu Y., Jiang C. (2024). Stretchable ionic-electronic bilayer hydrogel electronics enable in situ detection of solid-state epidermal biomarkers. Nat. Mater..

[135] Xiao M., Luo Y., Chen H., Wang H., Yao D., Wang D., Pan Y., Zha B., Yu Q., Xie R. (2025). Conformal Bioadhesive Hydrogel-Based Multimodal Wristband for Accurate, Mobile, and Real-Time Sign Language Translation and Human-Machine Interactions. Adv. Funct. Mater..

[136] Liu G., Wang D., Li H., Kong K., Xu K., Liu B., Wang R. (2025). Energy Dissipation Mediated by Multiple Noncovalent Interactions in Hydrogen-Bonded Organic Frameworks-Based Hydrogels for Wearable Gesture-to-Recognition Translation. Angew. Chem. Int. Ed..

